# Molecular regionalization of the developing amphioxus neural tube challenges major partitions of the vertebrate brain

**DOI:** 10.1371/journal.pbio.2001573

**Published:** 2017-04-19

**Authors:** Beatriz Albuixech-Crespo, Laura López-Blanch, Demian Burguera, Ignacio Maeso, Luisa Sánchez-Arrones, Juan Antonio Moreno-Bravo, Ildiko Somorjai, Juan Pascual-Anaya, Eduardo Puelles, Paola Bovolenta, Jordi Garcia-Fernàndez, Luis Puelles, Manuel Irimia, José Luis Ferran

**Affiliations:** 1 Department of Genetics, School of Biology, and Institut de Biomedicina (IBUB), University of Barcelona, Barcelona, Spain; 2 Centre for Genomic Regulation (CRG), Barcelona Institute of Science and Technology (BIST), Barcelona, Spain; 3 Universitat Pompeu Fabra (UPF), Barcelona, Spain; 4 Centro Andaluz de Biología del Desarrollo (CSIC/UPO/JA), Sevilla, Spain; 5 Centro de Biología Molecular Severo Ochoa CSIC-UAM and CIBERER, ISCIII, Madrid, Spain; 6 Instituto de Neurociencias, UMH-CSIC, Campus de San Juan, Sant Joan d'Alacant, Alicante, Spain; 7 The Scottish Oceans Institute, University of St Andrews, St Andrews, Fife, Scotland, United Kingdom; 8 Biomedical Sciences Research Complex, University of St Andrews, Fife, Scotland, United Kingdom; 9 Evolutionary Morphology Laboratory, RIKEN, Kobe, Japan; 10 Department of Human Anatomy and Psychobiology, School of Medicine, University of Murcia, Murcia, Spain; 11 Institute of Biomedical Research of Murcia (IMIB), Virgen de la Arrixaca University Hospital, University of Murcia, Murcia, Spain; California Institute of Technology, United States of America

## Abstract

All vertebrate brains develop following a common Bauplan defined by anteroposterior (AP) and dorsoventral (DV) subdivisions, characterized by largely conserved differential expression of gene markers. However, it is still unclear how this Bauplan originated during evolution. We studied the relative expression of 48 genes with key roles in vertebrate neural patterning in a representative amphioxus embryonic stage. Unlike nonchordates, amphioxus develops its central nervous system (CNS) from a neural plate that is homologous to that of vertebrates, allowing direct topological comparisons. The resulting genoarchitectonic model revealed that the amphioxus incipient neural tube is unexpectedly complex, consisting of several AP and DV molecular partitions. Strikingly, comparison with vertebrates indicates that the vertebrate thalamus, pretectum, and midbrain domains jointly correspond to a single amphioxus region, which we termed Di-Mesencephalic primordium (DiMes). This suggests that these domains have a common developmental and evolutionary origin, as supported by functional experiments manipulating secondary organizers in zebrafish and mice.

## Author summary

According to textbooks, vertebrate brains develop from a neural tube that rapidly becomes regionalized into the forebrain (which includes the secondary prosencephalon and diencephalon), midbrain, and hindbrain. These regions are then further subdivided; in particular, the diencephalon gives rise to the prethalamus, thalamus, and pretectum. However, embryological manipulations of brain signaling centers showed that the prethalamus behaves very differently than the thalamus and pretectum, which largely share their developmental potential with the midbrain. Therefore, this classic partition scheme might not be fully consistent from a developmental perspective. To better understand the origin and evolution of the regionalization of the vertebrate brain, we built a comprehensive molecular model of the incipient neural tube of amphioxus, an invertebrate chordate that shares multiple features with its vertebrate relatives. This model shows that the amphioxus nervous system is unexpectedly complex, sharing its basic blueprint with that of vertebrates. However, a single undivided region in amphioxus, which we termed Di-Mesencephalic primordium (DiMes), unambiguously corresponds to the region encompassing the thalamus, pretectum, and midbrain in vertebrates, indicating that these regions are also more closely related evolutionarily. Therefore, the diencephalon as a neuroanatomical compartment as well as the classic separation between forebrain and midbrain in vertebrates seem inconsistent from both an evolutionary and developmental perspective.

## Introduction

The vertebrate brain is arguably the most complex structure in nature. All vertebrates show a highly conserved construction plan, or Bauplan, of their central nervous system (CNS), which involves several major anatomical and genetic partitions and their subsequent subdivisions [1]. Understanding how this Bauplan has originated during evolution has been a matter of intense research and debate, but there is still no satisfactory answer. Do homologues to major vertebrate brain partitions exist in invertebrate species? Have new vertebrate partitions originated by subdivision and specialization of preexisting structures? Did positional genetic patterning mechanisms predate the origin of recognizable neuroanatomical regions, or did both originate concomitantly?

**Fig 1:**
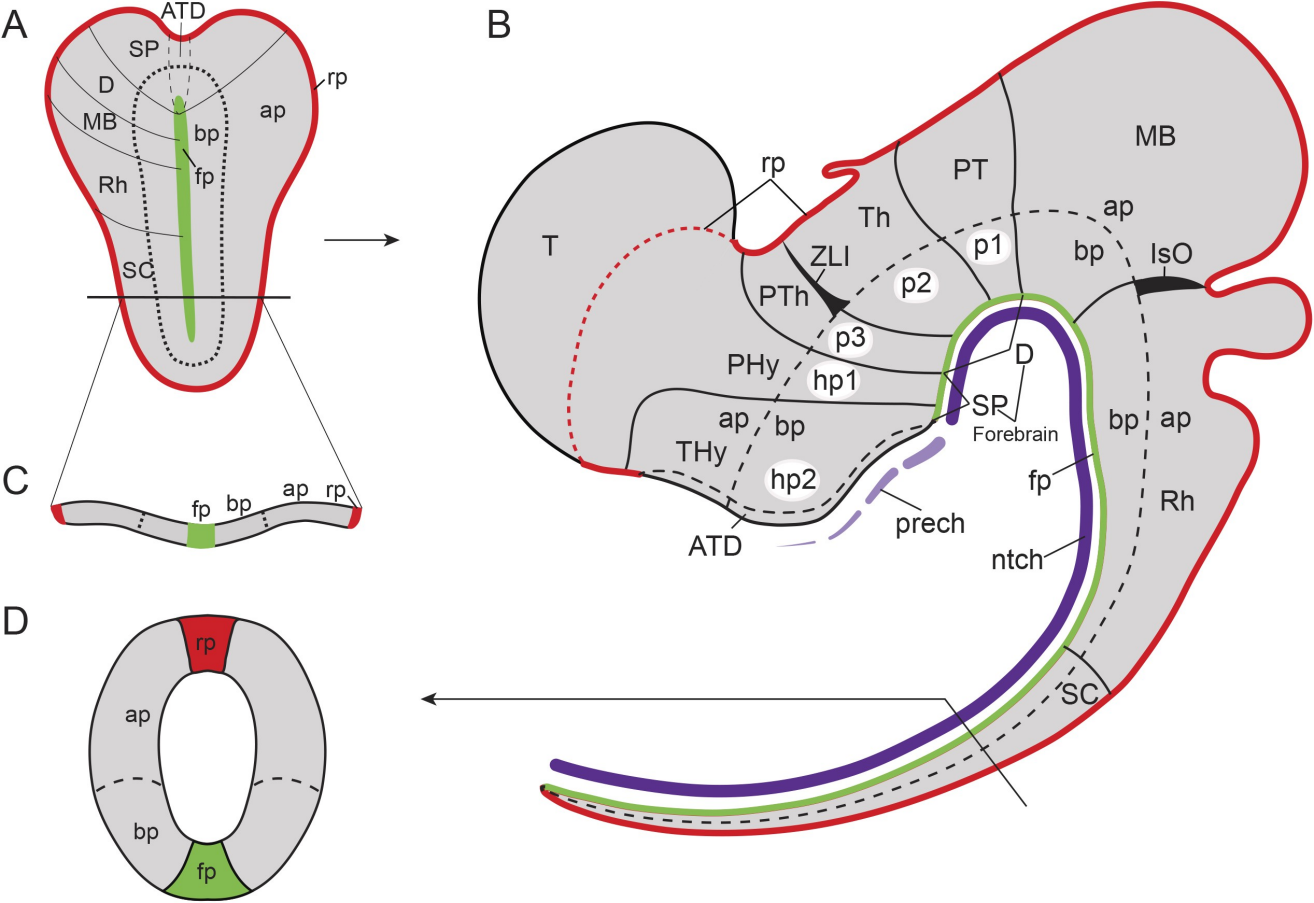
Bauplan of the vertebrate central nervous system (CNS). Schematic representation of the Bauplan of the vertebrate CNS according to the updated prosomeric model (Puelles and Rubenstein, 2015) at neural plate and early neural tube closure stages. **(A)** Schematic dorsal view of a representative neural plate of vertebrates depicting the components that define the longitudinal axis: floor plate (fp, in green) and parallel basal (bp), alar (ap) and roof (rp, in red) plates. Note that the fp does not reach the rostral boundary of the neural plate, whereas the bp, ap, and rp go around the fp; a similar phenomena can be observed in the caudal end of the neural plate. This peculiarity defines a rostral-most dorsoventral (DV) region termed acroterminal domain (ATD). From this domain, several anteroposterior (AP) partitions can be defined molecularly from rostral to caudal: secondary prosencephalon (SP) and diencephalon proper (D) (classic forebrain components), midbrain (MB), rhombencephalon (Rh), and spinal cord (SC) regions. **(B)** Schematic lateral view of an early closed neural tube in a more advanced stage of regionalization. Longitudinal components are indicated as fp (green), bp, ap, and rp (red). At this stage, the vertebrate neural tube is characterized by several definitive neuromeric units (i.e., AP partitions with all the DV components). The forebrain is subdivided into SP, with peduncular and terminal prosomeres (hp1 and hp2, respectively), which include the hypothalamic region, telencephalon (Tel), and the optic vesicles, and D (with prosomeres 1, 2, and 3 [p1–p3], which are represented at the ap level by pretectum [PT], thalamus [Th], and prethalamus [PTh], respectively). More caudally, MB, Rh, and SC regions are identified (for simplicity, their respective neuromeric components are not depicted). The secondary organizers zona limitans intrathalamica (ZLI) and the isthmic organizer (IsO) are located between the PTh and Th regions, and the MB and Rh regions, respectively. **(C)** The transversal section throughout the open neural plate in **(A)**, showing the main longitudinal components (fp, bp, ap, and rp) arranged medio-laterally at this stage. **(D)** Cross section through the closed neural tube, showing the DV relationships of the same longitudinal components highlighted in (**C**).

These and related questions have been investigated mainly from an evolutionary developmental (Evo-Devo) perspective, since early developing brains have not yet undergone complex morphogenetic deformations and are thus more amenable to evolutionary comparisons between distantly related species. In the case of vertebrates, the CNS arises very early in embryonic development via neural induction. The neuroectodermal plate represents the earliest CNS primordium, which then folds into a closed tube during neurulation. Already at neural plate stages, the CNS becomes regionalized molecularly into large anteroposterior (AP) regions. According to the prosomeric model [2–4], this Bauplan includes the secondary prosencephalon and diencephalon proper (forebrain), midbrain, hindbrain, and spinal cord (Fig 1A and 1B). These primary regions are further partitioned into smaller transverse AP units, identified as brain segments or neuromeres (Fig 1B). Two lineal neuroepithelial signal sources known as **secondary organizers** are crucial for this process: the zona limitans intrathalamica (ZLI or mid-diencephalic organizer) and the isthmic organizer (IsO, located in the Midbrain–Hindbrain Boundary, MHB; Fig 1B). These organizers are characterized by the release of diffused morphogen signals (SHH and FGF8/WNT1, respectively) and are involved in AP regionalization and differential specification of the diencephalic, mesencephalic, and some rostral rhombencephalic neuromeres [5–13].

**Fig 2:**
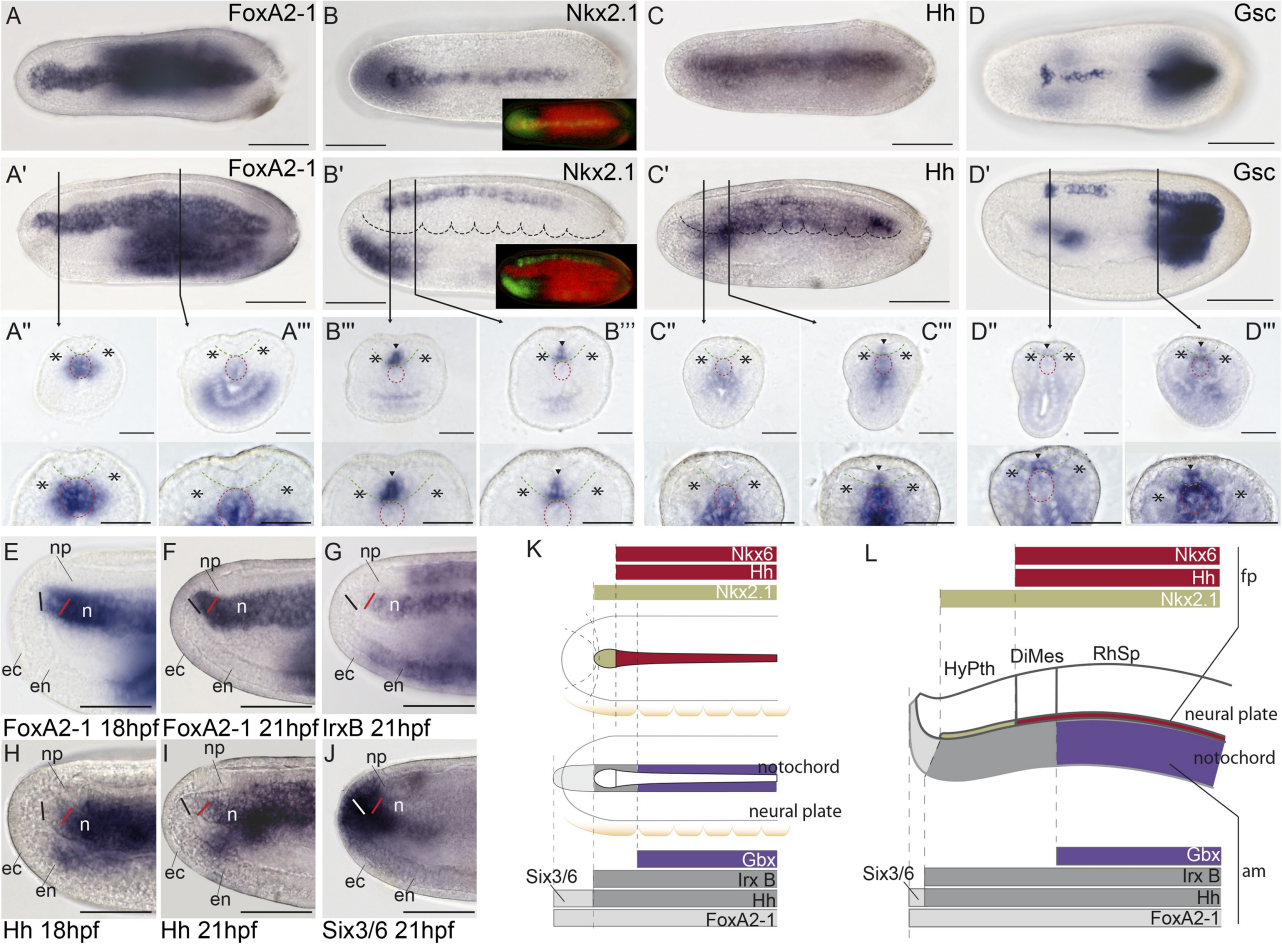
Molecular regionalization of the amphioxus floor plate and axial mesoderm. **(A-A′′′)** *FoxA2-1* is expressed throughout the notochord, as shown by whole-mount in situ hybridization in dorsal **(A)** and lateral **(A′)** views and in situ hybridization in cryostat transversal sections **(A′′,A′′′)**. (**B-B′′′)** *Nk2*.*1* mRNA is expressed throughout the entire floor plate, as observed in dorsal **(B)** and lateral **(B′)** views, and in cryostat transversal sections (**B′′,B′′′**). Insets in (**B**) and (**B′**) show the combined *FoxA2-1* and *Nkx2*.*1* expression patterns using pseudocolors, indicating that *Nkx2*.*1* is expressed above the notochord but does not reach its rostral boundary. (**C-C′′′**) *Hh* mRNA is detected in most of the floor plate, with exception of the rostral-most portion (hypothalamo-prethalamic primordium [HyPTh] floor plate) as observed in dorsal **(C)** and lateral **(C')** views, and in cryostat transversal sections (**C′′, C′′′**)**. (D-D′′′)** *Gsc* expression is observed in different rostro-caudal patches in dorsal **(D)** and lateral **(D′)** views and in cryostat transversal sections **(D′′, D′′′)**. **E-J)** Detailed analysis of the rostral end of the notochord using whole-mount data at 18 h post fertilization (hpf) and 21 hpf stages further supports that *FoxA2-1* **(E,F)** is present throughout the entire length of the notochord; *IrxB* **(G)** and *Hh* **(H,I)** are absent rostrally and expressed caudally, and *Six3/6* **(I)** is expressed only in the rostral tip. (**K,L**) The rostral molecular code (*Six3/6* and *FoxA2-1* positive, but *IrxB* and *Hh* negative) is summarized in dorsal (**K**) and lateral (**L**) schematic representations. In cryostat sections, asterisks mark somites, and arrowheads indicate neural expression; red and green dotted lines delineate the notochord and neural plate, respectively. Abbreviations: np, neural plate; ec, ectoderm; en, endoderm; n, notochord; fp, floor plate; am, axial mesoderm. Scale bar: 50 μm.

Furthermore, along the neural tube, each neuromere is composed of four continuous dorsoventral (DV) domains: roof, alar, basal, and floor plate regions (Fig 1A–1D). Importantly, the prospective DV pattern is already observed at neural plate stages, corresponding to its mediolateral dimension (Fig 1A, 1C and 1D): the future floor corresponds to the neural plate midline, whereas the future roof lies at the border of the neural plate. The rostral end of the neural plate is thus morphologically singular because the floor does not reach the anterior border of the plate but ends rostrally at the prospective mamillary hypothalamic region, in coincidence with the underlying rostral tip of the notochord [14]; therefore, the roof, alar, and basal plates concentrically cross the midline at the terminal wall (future acroterminal domain), curving around the rostral end of the floor plate (Fig 1A, [2,4,15]).

An important breakthrough in the study of comparative neuroanatomy and the evolutionary origin of CNSs has been the observation that each established AP and DV anatomical partition in a given species is characterized by the differential expression of specific gene markers early in development in a combinatorial code that we refer to as genoarchitecture [14]. These molecular codes create clear-cut molecular boundaries between the neuromeres, and often correspond with visible external bulges due to the differential proliferation of the progenitors because of their distinct genoarchitectonic profiles [3,15]. Strikingly, the number of neuromeric units and their associated genoarchitecture is highly conserved in all vertebrate groups, including the basal-branching agnathans [16–34]. This implies that a fundamentally conserved anatomical CNS Bauplan and its corresponding genetic blueprint have existed at least since the last common ancestor of vertebrates.

Therefore, a major approach to understanding the origins of this Bauplan has been to investigate the expression of orthologs of key gene markers in chordate and nonchordate invertebrate species. Remarkably, a subset of these markers show fixed relative AP positions, suggesting that some of the regional genoarchitectonic codes of vertebrates were established prior to the origin of the vertebrate brain Bauplan. For example, the transverse genetic boundaries defined by the abutting expression of *Fezf*/*Irx* and *Otx*/*Gbx*—which in vertebrates correspond to the anatomical positions in which the ZLI and IsO secondary organizers will develop, respectively—are observed in the CNSs of species as diverged as amphioxus and fruit flies [35–37]; although, these sites lack the expression of the morphogens responsible for the organizer activity in vertebrates [38–41]. Moreover, some markers expressed in the annelid *Platynereis durmeilii* show remarkable topologic similarity with the mediolateral and AP molecular pattern in vertebrates [42–44]. In one of the most striking cases of genetic patterning conservation observed between vertebrates and invertebrates, the diffuse epidermal nervous system of hemichordates displays multiple vertebrate-like AP genetic codes, including a ZLI-like domain with equivalent relative expression of *hh*, *six3*, *fng*, *otx*, and *wnt8* orthologs and an IsO-like region coexpressing *fgf8/17/18* and *wnt1*, suggesting conservation of the underlying genetic programs despite the fact that they are patterning divergent structures in the two lineages [45–48].

Altogether, these studies thus suggest that multiple defining genetic programs that pattern the vertebrate brain predate its evolutionary emergence. However, the major limitation of these nonchordate model systems to investigate the origin of the vertebrate brain Bauplan is the lack of an unambiguous anatomical and topological reference system. Even under the assumption that the nervous systems of these invertebrate phyla are truly homologous to the vertebrate CNS, each one has its own set of clade-specific characters and thus correspond to different variational modalities of CNSs [49]. This impedes direct topological comparisons, leaving similarities of gene expression patterns as the only support for any hypothesized homology assignment. For this reason, the cephalochordate amphioxus has traditionally been the most studied invertebrate species for comparative analyses with vertebrates. Unlike nonchordates, amphioxus develops its tubular CNS from a neural plate in the same way that vertebrates do, thus allowing direct topological comparisons of prospective brain regions. Furthermore, unlike tunicates, cephalochordates have undergone slow evolutionary rates, both genomically and morphologically [50,51]. Multiple studies on this organism have shown, for instance, that the *Otx/Gbx* and *Fez/Irx* genetic boundaries [36,37,52] as well as part of its neural *Hox* AP patterning [53–57] are conserved with vertebrates. Similarly, orthologs of many other key vertebrate genes have been implicated in neural function and development in amphioxus (see S1 Table for a list of previously described gene expression patterns in amphioxus with relevance to CNS development that have been used in this study). These reports, together with multiple comprehensive and integrating reviews [41,58–64], have provided important insights on the presence of molecularly-defined partitions in the developing amphioxus CNS. Nonetheless, these studies have been performed by different research groups, using different amphioxus species, and usually focused on the expression of a single gene at multiple embryonic stages. This has made the systematic integration and accurate combinatorial analyses of these expression patterns a complex task.

To address these difficulties, we mapped here 48 genes with well-known roles in vertebrate CNS patterning on a single amphioxus developmental stage, the 7-somite mid-neurula, in which a wide spectrum of orthologs of vertebrate neural gene markers is expressed. With these data, we propose an integrative model of the molecular regionalization of the amphioxus developing CNS that is consistent and comparable with the prosomeric model of the vertebrate CNS Bauplan. Our results show that, at the mid-neurula stage, the amphioxus CNS primordium has an unexpectedly complex genoarchitecture, with three major molecularly distinguishable AP divisions (and some secondary subdivisions) and a set of standard DV zones. Strikingly, direct topological comparison between the molecular models of the two lineages, as well as extensive novel and previously reported functional data, suggest that the vertebrate territory comprising the diencephalic neuromeric units corresponding to thalamus and pretectum (prosomeres p2, p1), but not the prethalamus (p3), share with the midbrain a common ontogenetic and evolutionary origin, and, altogether, are homologous to a nonregionalized *Pax4/6*-positive domain in amphioxus, which we termed Diencephalo-Mesencephalic primordium (DiMes). Whether resulting from an increase in complexity in vertebrates or, alternatively, a simplification in amphioxus compared to the last common ancestor of chordates, these results suggest that the differences in AP Bauplan complexity between the two lineages are likely linked to the secondary organizers of vertebrates (ZLI and IsO), which are absent in amphioxus. Experimental abrogation and manipulation of these organizers in vertebrate species generate phenotypic defects that are consistent with this hypothesis.

## Results

### Molecular markers define and regionalize the amphioxus floor plate

AP and DV subdivisions in developing chordate neural tubes are defined according to axial references. Conventionally, such references are provided in vertebrates by the axial mesoderm (the notochord), the floor plate, roof plate, and alar–basal boundary within the lateral walls of the neural tube, all of which are topologically parallel to each other (Fig 1). Amphioxus has a notochord, which extends singularly beyond the forebrain [65], and a floor plate [66–70]. As previously reported for the Floridan amphioxus *Branchiostoma floridae* [71,72], we observed in the European amphioxus *B*. *lanceolatum* that the gene *FoxA2-1* is a selective marker of the notochord (Fig 2A–2A′′′, 2E and 2F), while *Nkx2*.*1* seems to be a general floor plate marker at the 7-somite neurula stage (Fig 2B–2B′′′). As in vertebrates, in which its expression in the floor plate is transient [73], *Nkx2*.*1* expression is highly dynamic during amphioxus CNS development (S1 Fig). *Nkx2*.*1* is observed along the entire presumptive floor plate at early- and mid-larval stages, but it subsequently becomes restricted rostralwards.

Since the whole neural tube of amphioxus sits on top of the notochord, it should be, in theory, regarded as topographically epichordal. Thus, as the floor plate is induced vertically by the notochord [74–76], we a priori expected the amphioxus floor plate to extend all along the acroterminal neural midline (up to the neuropore), in contrast to the vertebrate floor plate, which stops at the mamillary pouch of the hypothalamus, coinciding with the approximate position of the rostral tip of the notochord (Fig 1A and 1B; [2,4]). Instead, we observed that the floor plate, defined by *Nkx2*.*1* expression, does not reach the anterior neural border, but it ends in a slightly expanded median patch that recalls the mamillary hypothalamic ending observed in vertebrates (Fig 2B and 2B′ insets, K; see also [66]). Interestingly, *Hedgehog (Hh)*, which is a well-established floor plate marker in vertebrates [74,77,78], and *Nkx6* are also expressed in the amphioxus floor plate, but their anterior limit of expression is more caudal than that of *Nkx2*.*1* (Figs 2C–2C′′′, 2K, 2L and 7D–7D′′; a similar expression for *Hh* has been reported in *B*. *floridae* [69]). *Goosecoid* (*Gsc*) is also expressed in the floor plate (in contrast to previous reports [79]) in a variable and patchy pattern that might reflect cyclic dynamic changes (Fig 2D–2D′′′). These markers differentiate two major floor plate AP regions: (i) a rostral-most median floor domain characterized by only *Nkx2*.*1* expression, which corresponds to the floor plate of the forebrain region that we refer to as the amphioxus hypothalamo-prethalamic primordium (HyPTh; see below and Fig 2K and 2L); and (ii) the rest of the floor plate, defined by *Hh*, *Gsc*, *Nkx6*, and *Nkx2*.*1* expression.

### Molecular heterogeneity of the amphioxus axial mesoderm: Notochord and a possible prechordal primordium

We next examined the genoarchitecture of the amphioxus axial mesoderm to assess the existence of a putative prechordal plate homolog. According to the updated prosomeric model [2,4], the latter tissue lies topologically rostral to the neural primordium and the notochord (Fig 1B). As mentioned above, *FoxA2-1* labels the whole amphioxus prospective notochord (Fig 2A–2A′′′). On the other hand, the expression of both *Hh* and *IrxB* in the axial notochordal tissue does not reach the rostral tip of the *FoxA2-1*–positive domain, stopping beneath the rostral end of the *Nkx2*.*1*–positive HyPTh floor plate (Fig 2L, 2E–2J; it should be noted, however, that *IrxB* expression seems to reach the anterior tip of the notochord in *B*. *floridae* [80]). Moreover, in the amphioxus axial mesoderm, *Six3/6* expression was observed exclusively in the rostral tip of the *FoxA2-1*–positive domain, beyond the *Hh/IrxB*–positive part of the notochord (Fig 2K, 2L and 2J; see also [81]). Interestingly, this *Six3/6* expression is maintained at later stages, when the notochord is fully formed [81], indicating that its rostral tip has a distinct molecular signature compared to the rest of the notochord. Remarkably, in vertebrates, *Six3* is expressed in the prechordal plate but not in the notochord at any level [24]; therefore, the rostral notochordal tip of amphioxus might represent a possible prechordal plate homologue, previously unrecognized due to its histologic similarity to the notochord proper (see Discussion).

**Fig 3:**
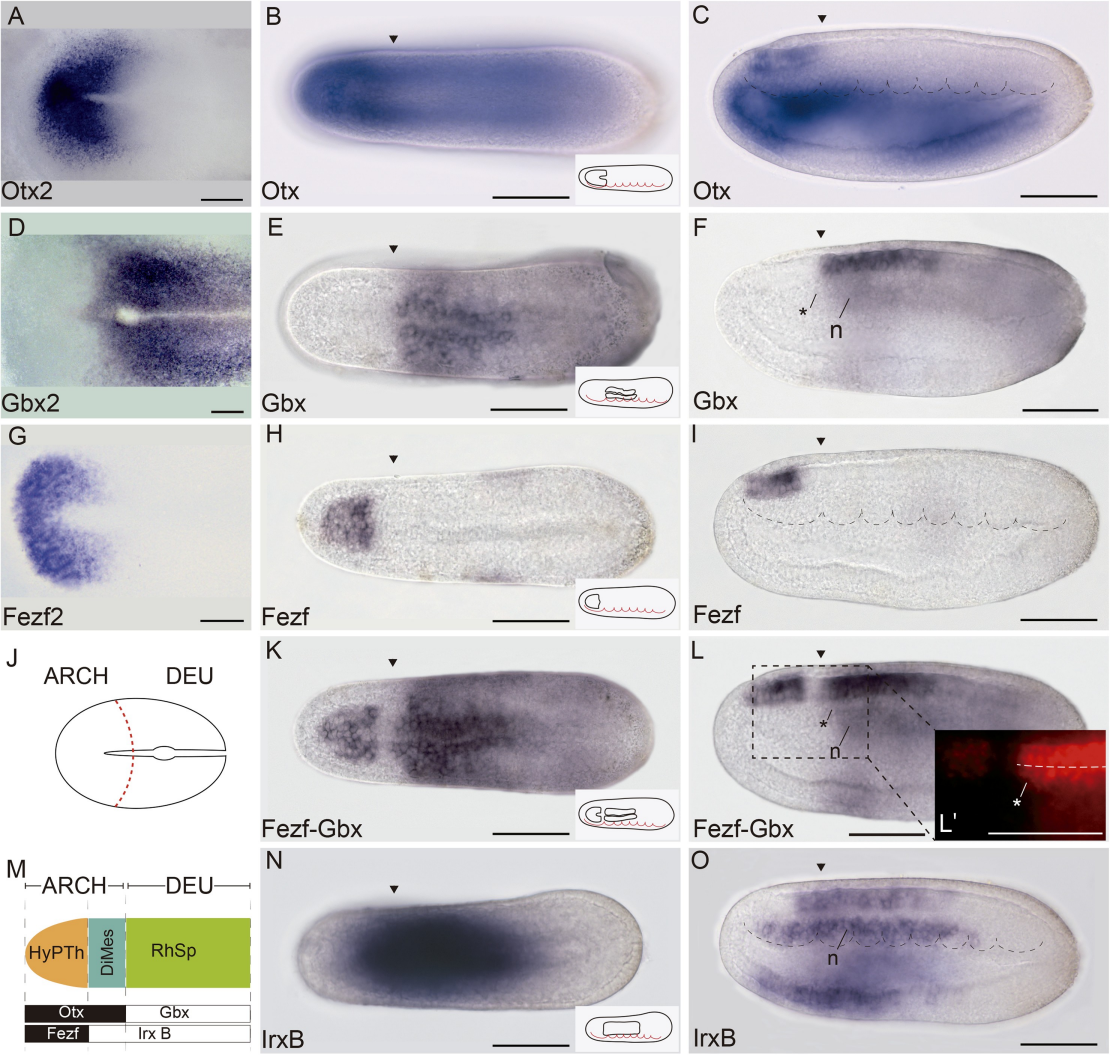
Three major molecular anteroposterior (AP) regions are observed in the incipient amphioxus neural tube. **(A)** Whole-mount in situ hybridization of chicken *Otx2* at Hamburger–Hamilton 5 (HH5) stage. (**B,C**) Expression of amphioxus *Otx* at 21 h post fertilization (hpf) in dorsal **(B)** and lateral **(C)** views. (**D)** Whole-mount in situ hybridization of chicken *Gbx2* at HH5 stage. (**E,F**) Expression of amphioxus *Gbx* at 21 hpf in dorsal **(E)** and lateral **(F)** views. **G)** Whole-mount in situ hybridization of chicken *Fezf2* at HH5 stage. (**H,I**) Expression of amphioxus *Otx* at 21 hpf in dorsal **(H)** and lateral **(I)** views. (**J)** Schematic representation of HH5 chicken neural plate with the archencephalic prototagma (ARCH) and deuteroencephalic prototagma (DEU) domains depicted. The boundary between ARCH and DEU correspond to the border between *Otx2* and *Gbx2* expression patterns **(A,D)**. (**K,L)** Double chromogenic in situ hybridization combining amphioxus *Fezf* and *Gbx* probes in dorsal **(K)** and lateral **(L)** views, showing two subdivisions in the amphioxus ARCH territory: a rostral hypothalamo-prethalamic primordium (HyPTh) domain (*Fezf* and *Otx* positive) and a caudal Di-Mesencephalic primordium (DiMes) domain (*Fezf negative* and *Otx* positive) **(L′)**. (**M**) Schematic representation of the three major AP subdivisions in the amphioxus central nervous system (CNS) at the 21 hpf stage and the relative expression of their key markers. (**N,O**) Single chromogenic in situ hybridization with an amphioxus *IrxB* probe in dorsal **(N)** an lateral **(O)** views. Insets in B, E, H, K, and N depict the neural components of the corresponding gene expression patterns. Arrowheads mark the ARCH–DEU boundary, and asterisks mark the corresponding limit at the notochord level, based on *Gbx* expression. Abbreviations: n, notochord. Scale bar: 50 μm.

Finally, we found that *Gbx* expression appears restricted to a more caudal sector of the notochord, whose rostral border is posterior to the caudal boundary of the HyPTh neural domain (Figs 2K, 2L, 3F and 3L inset). Previous studies [37] and other observations described below suggest that the *Gbx*-expressing domain of the notochord and overlying neural tissue begins at the rostral end of the major region we term Rhombencephalo-Spinal primordium (RhSp; Fig 2L). In summary, we observed that the amphioxus axial mesoderm is subdivided molecularly into various regions, which have direct correspondence with major subdivisions in the overlaying neural plate.

### The incipient neural tube of amphioxus possesses distinct floor, basal, and alar plates

**Fig 4:**
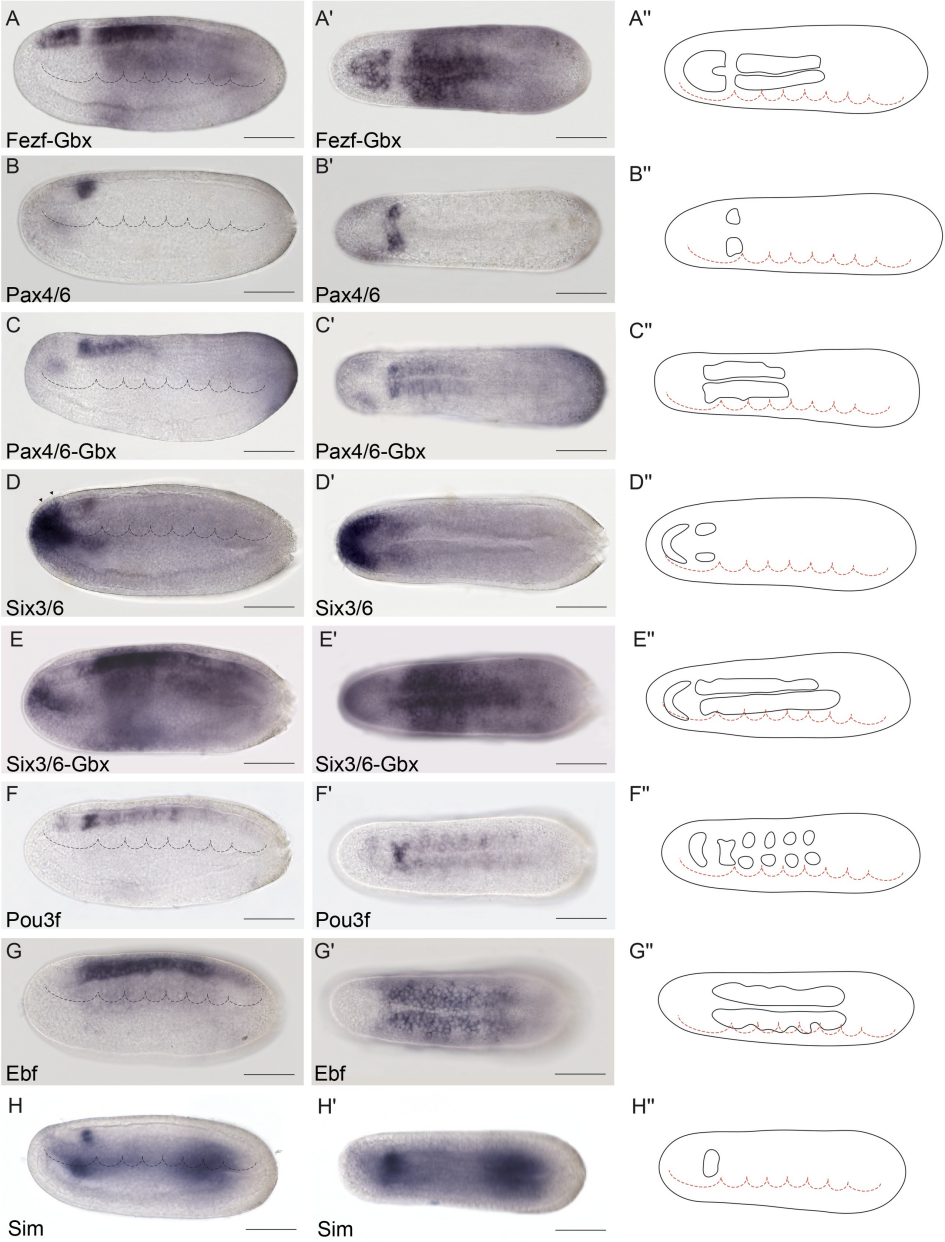
Genoarchitectonic signature of the Di-Mesencephalic primordium (DiMes). **(A-A′′)** Combined *Fezf-Gbx* expression defines a gap of expression in the caudal archencephalic prototagma (ARCH), identified as the DiMes (as per Fig 3K and 3L, for reference). **(B-E′′)** Whole-mount chromogenic in situ hybridization of *Pax4/6* **(B-B′′)** or *Six3/6* **(D-D′′)** alone or each one combined with *Gbx* in a double in situ hybridization **(C-C′′** and **E′-E′′**, respectively) reveal that both genes are expressed in the DiMes domain. The two arrowheads in **(D)** indicate the expression of *Six3/6* in Rostral-hypothalamo–prethalamic primordium (Rostral-HyPTh). (**F-F′′**) *Pou3f* is highly expressed in DiMes but with a decreased signal in the Rostral-HyPTh and Intermediate-HyPTh primordia and in some areas of the deuteroencephalic prototagma (DEU). (**G-G′′)** *Ebf* mRNA was detected in the DiMes and DEU domains. (**H-H′′)** *Sim* neural expression was observed exclusively in the DiMes domain at the analyzed stage. Expression patterns correspond to lateral **(A-H)** or dorsal views **(A′-H′)** at the 21 h post fertilization (hpf) embryonic stage and are represented in schematics dorsal views **(A′′-H′′)**. Somites (dotted lines) were used as main landmarks to localize the position of the patterns analyzed in the late neural plate. Scale bar: 50 μm.

**Fig 5:**
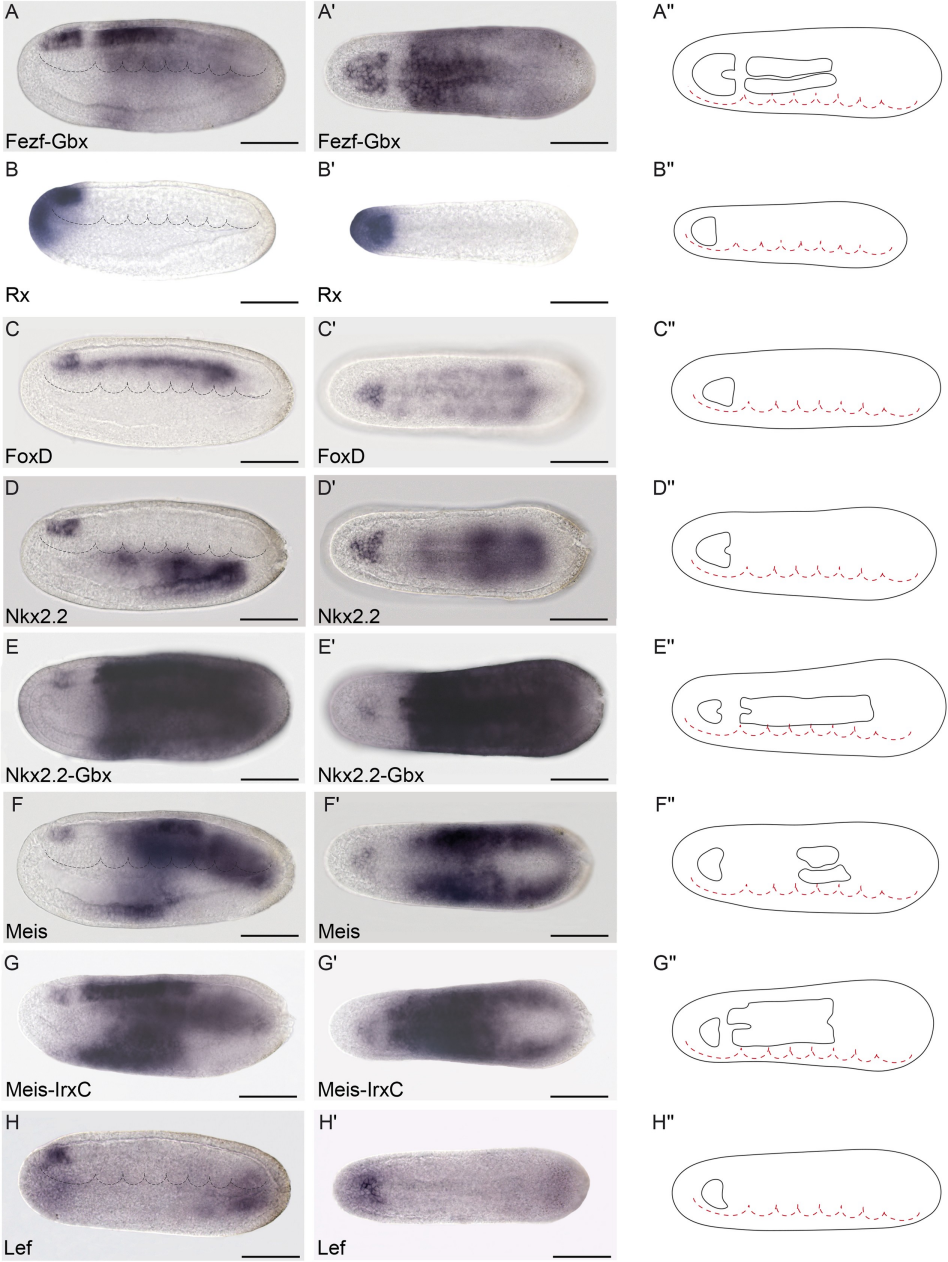
Genoarchitectonic signatures of the hypothalamo-prethalamic primordium (HyPTh) (I). **(A-A′′)** Combined *Fezf-Gbx* expression defines a *Fezf*-positive rostral archencephalic prototagma (ARCH) territory identified as HyPTh (as per Fig 3K and 3L, for reference). (**B-B′′**) *Rx* is specifically expressed throughout the entire HyPTh primordium. (**C-C′′**) Neural expression of *FoxD* was detected only in the basal and floor plates of HyPTh. (**D-E′′**) *Nkx2*.*2* is expressed in the Rostral-HyPTh and Interm-HyPTh domains but not in Caudal-HyPTh (D-D′′), as observed by a large gap in a double in situ hybridization for *Nkx2*.*2* and *Gbx* **(E-E′′). (F-G′′)** Similarly, *Meis* mRNA is only detected in the basal plate of the Rostral and Intermediate domains of HyPTh **(F-F′′)**, leaving a gap of expression when combined with *IrxC* **(G-G′′).** Further expression of *Meis* is also detected in specific deuteroencephalic prototagma (DEU) areas **(F-F′′)**. (**H-H′′)** *Lef* is expressed in the basal plate of Rostral-HyPTh and Interm-HyPTh. Expression patterns correspond to lateral **(A-H)** or dorsal views **(A′-H′)** at the 21 h post fertilization (hpf) embryonic stage and are represented in schematics dorsal views **(A′′-H′′)**. Somites (dotted lines) were used as main landmarks to localize the position of the patterns analyzed in the late neural plate. Scale bar: 50 μm.

**Fig 6:**
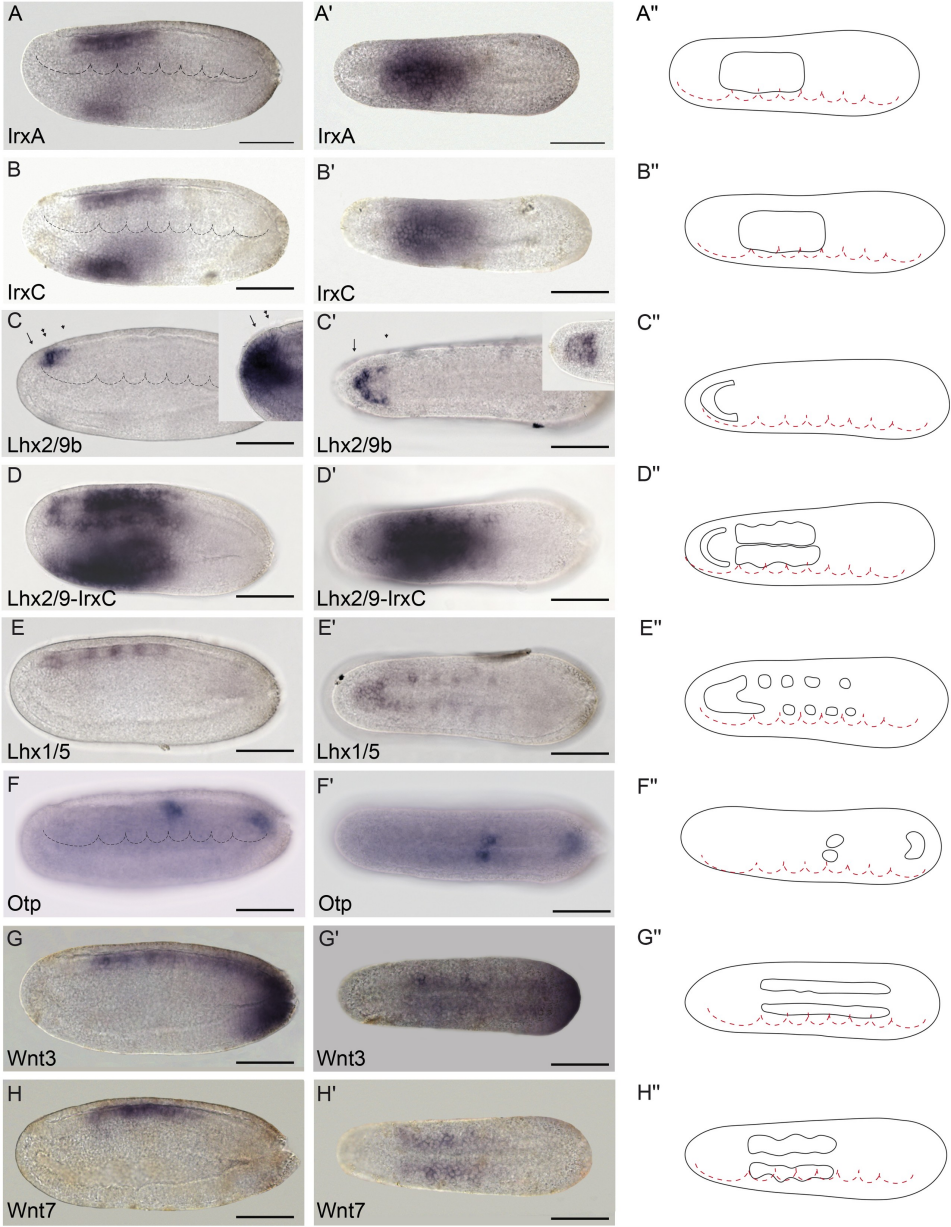
Genoarchitectonic signatures of the hypothalamo-prethalamic primordium (HyPTh) (II). **(A-A′′)** *IrxA* mRNA expression is observed from the HyPTh/Di-Mesencephalic primordium (DiMes) boundary extending caudally to the rostral portion of the Rhombencephalo-Spinal primordium (RhSp) domain. (**B-B′′)** *IrxC* mRNA expression is observed from the HyPTh/DiMes boundary, extending caudally to the rostral portion of the RhSp domain. (**C-D′′)** *Lhx2/9b* marks the alar plate in the Rostral-HyPTh and Intermediate-HyPTh domains **(C-C′′)**, as shown by a gap of expression in a double in situ hybridization between *Lhx2/9b* and *IrxC* **(D-D′′)**. The lateral view of *Six3/6* expression (inset in C) is provided for comparison with *Lhx2/9b* and highlights the restricted expression of *Six3/6* to the Rostral-HyPTh (compare the region between the arrow and the single arrowhead, which corresponds to the Rostral-HyPTh and Intermediate-HyPTh domains, with the region between the arrow and double arrowhead, which includes only the Rostral-HyPTh domain **(C,C′)**). On the other hand, a dorsal view of *Fezf* (inset in C′**)** shows expression across both the alar and basal plates of the HyPTh. (**E,E′′)** *Lhx1/5* mRNA expression is observed only in the basal plate of the HyPTh and DiMes primordia and in some RhSp subdivisions (see inset in C′ for comparison). (**F-F′′)** *Otp* is a key hypothalamic marker in vertebrates but was only found in amphioxus in one domain at the RhSp region. (**G-H′′)** *Wnt3* and *Wnt7* mRNAs were detected from the DiMes/RhSp border, extending caudally in the entire RhSp region. Expression patterns correspond to lateral **(A-G)** or dorsal views **(A′-G′)** at the 21 h post fertilization (hpf) embryonic stage and are represented in schematics dorsal views **(A′′-G′′)**. Somites (dotted lines) were used as main landmarks to localize the position of the patterns analyzed in the late neural plate. Scale bar: 50 μm.

**Fig 7:**
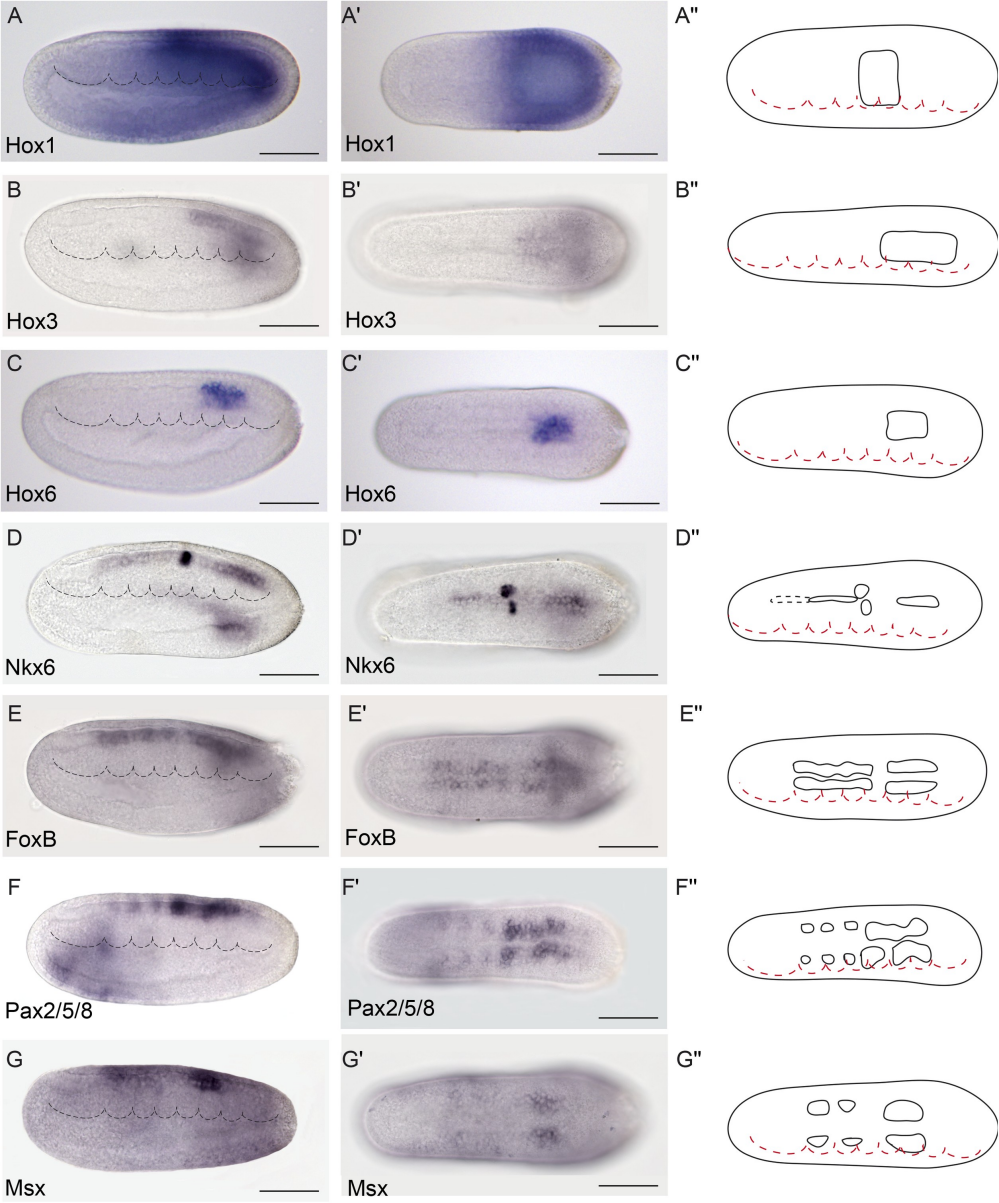
Genoarchitectonic signatures of the Rhombencephalo-Spinal primordium (RhSp). **(A-C′′)**. *Hox1*, *Hox3*, and *Hox6* were expressed in the alar and basal plates of some caudal domains of the RhSp region in a sequential rostro-caudal order. (**D-D′′)** *Nkx6* was detected at different degrees of expression mainly at the floor plate of the Di-Mesencephalic primordium (DiMes) and RhSp domains and a localized bilateral spot at the equivalent position of the fifth somite. (**E-E´´)** *FoxB* mRNA was observed extending caudally from the DiMes/RhSp border into the basal plate of the entire RhSp region. **(F-G´´)** *Pax2/5/8* and *Msx* mRNAs were detected in some patches in the alar plate of the RhSp region. Expression patterns correspond to lateral **(A-G)** or dorsal views **(A′-G′)** at the 21 h post fertilization (hpf) embryonic stage, and are represented in schematics dorsal views **(A′′-G′′)**. Somites (dotted lines) were used as main landmarks to localize the position of the patterns analyzed in the late neural plate. Scale bar: 50 μm.

**Fig 8:**
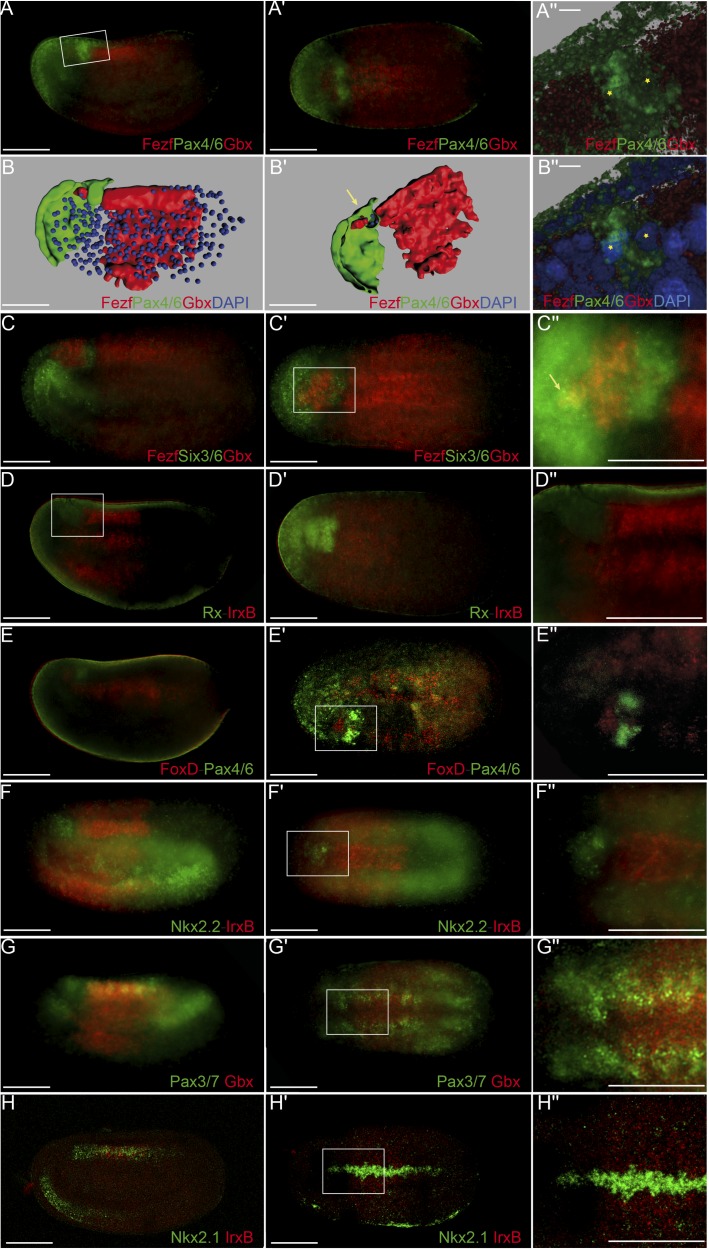
Precise genetic boundaries define three major anteroposterior (AP) partitions. **(A-B′′)** Triple fluorescent in situ hybridization combining *Fezf-Pax4/6-Gbx* in lateral **(A)** and dorsal views **(A′)** reveals clear-cut boundaries between the hypothalamo–prethalamic primordium (HyPTh) *Fezf*+, the Di-Mesencephalic primordium (DiMes) *Pax4/6*+, and the Rhombencephalo-Spinal primordium (RhSp) *Gbx*+ regions; details are summarized in a 3-D reconstruction **(B,B′)**. A magnified view of *Pax4/6* expression combined with DAPI showed that the DiMes domain consists of two rows of cells along the AP axis (asterisks)**(A′′,B′′). (C-C′′)** Triple fluorescent in situ hybridization combining *Fezf-Six3/6-Gbx* probes in lateral **(C)** and dorsal views **(C′)** confirms a rostral *Fezf*+ domain (HyPTh), a caudal *Gbx*+ domain (RhSp), and a double-negative domain in between characterized by *Six3/6* expression (DiMes). A magnified view **(C′′)** helps to visualize a rostral domain with *Six3/6* and *Fezf* coexpression (yellow staining, arrow) that we identified as the Rostral-HyPTh domain. (**D-D′′)** Double fluorescent in situ hybridization combining *Rx* and *IrxB* probes in lateral **(D)** and dorsal views **(D´)** show that *Rx* is expressed in the entire HyPTh territory, stopping caudally at the HyPTh/DiMes boundary; details can be observed in the magnified view **(D′′)**. **(E-E′′)** Double fluorescent in situ hybridization combining *FoxD* and *Pax4/6* probes in lateral **(E)**, dorsal **(E′),** and magnified dorsal **(E′′)** views shows that the small territory expressing *FoxD* corresponds to the basal plate of the entire HyPTh primordium, stopping caudally at the DiMes border. (**F-F′′)** Double fluorescent in situ hybridization combining *Nk2*.*2* and *IrxB* probes in lateral **(F),** dorsal **(F′),** and magnified dorsal **(F′′)** views determines that *Nk2*.*2* is expressed only in the alar and basal plate of the Rostral-HyPTh and Intermediate-HyPTh domains, leaving a negative gap corresponding to the Caudal-HyPTh domain. **(G-G′)** Double fluorescent in situ hybridization combining *Pax3/7* and *Gbx* probes identifies patches of *Pax3/7* expression in the Caudal-HyPTh domain. (**H,H′)** Double fluorescent in situ hybridization combining *Nkx2*.*1* and *IrxB* probes shows that *Nk2*.*1* expression in the floor plate extends rostrally beyond the HyPTh/DiMes boundary. Scale bar in A-H′: 50 μm except A′′ and B′′, scale bar: 5 μm.

**Fig 9:**
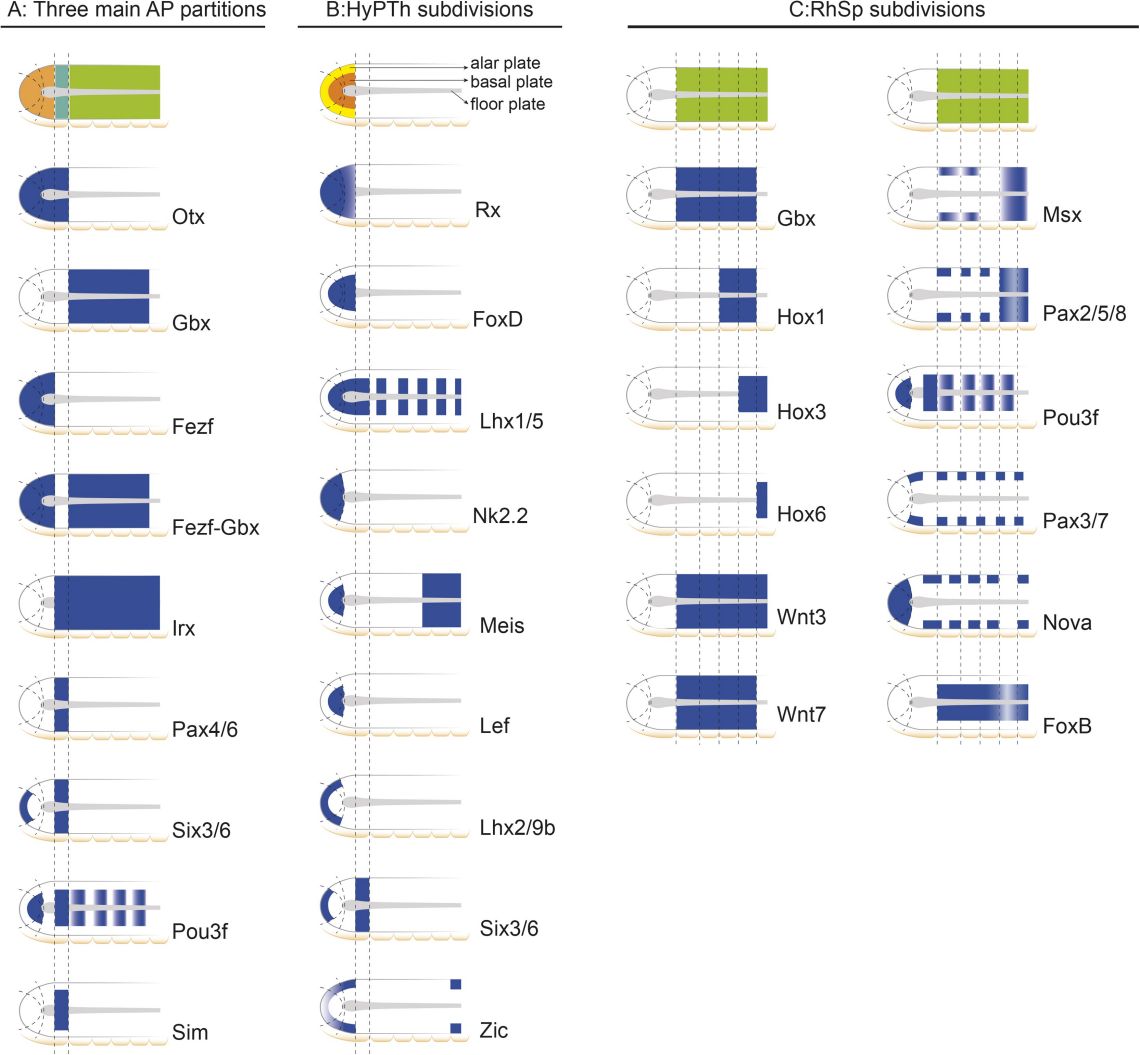
Schematic representation of informative markers used in this study. **(A)** Main tagmata and Hypothalamo-Prethalamic (HyPTh), Di-Mesencephalic (DiMes), and Rhombencephalo-Spinal (RhSp) primordia. **(B)** HyPTh internal subdivisions. (**C)** RhSp internal subdivisions.

**Fig 10:**
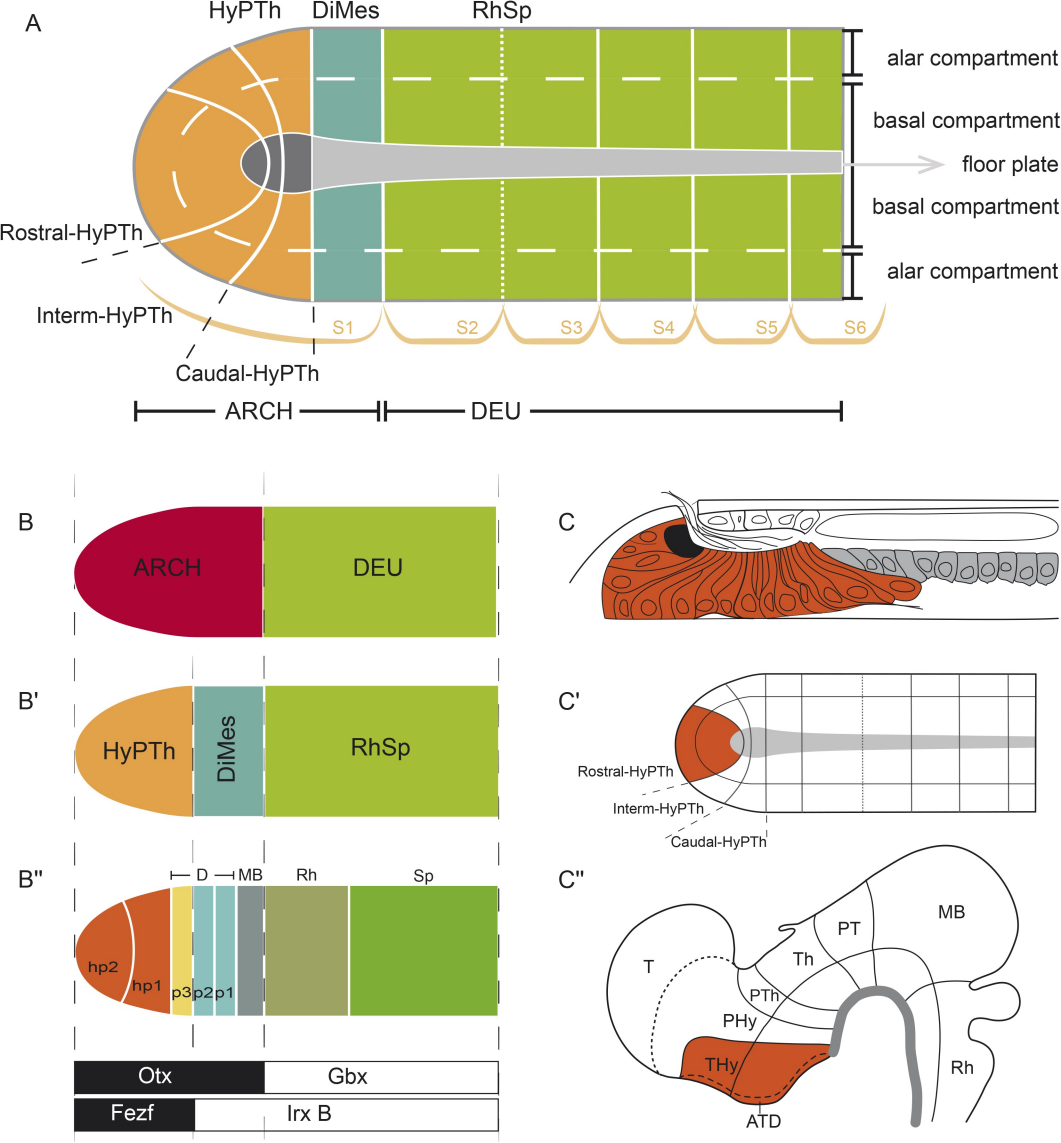
Genoarchitectonic model of the developing central nervous system (CNS) at the amphioxus 7-somite neurula stage. **(A**) Summary of all identified anteroposterior (AP) and dorsoventral (DV) partitions of the neural plate of amphioxus. (**B-B′′**) Topological comparison of major molecular subdivisions between cephalochordates and vertebrates. (**C,C′**) Neural plate model highlighting the basal and alar plates of Rostral-hypothalamo–prethalamic primordium (Rostral-HyPTh) (orange) and the whole floor plate domain (gray) and its correspondence in a late larval stage (adapted from [90]). (**C′′**) Vertebrate neural tube highlighting the Terminal-Hypothalamic prosomere (orange) and the whole floor plate (grey).

Previous gene expression studies provided evidence for the presence of longitudinal zones positioned parallel to the floor plate, implying DV patterning in the amphioxus CNS [69,82]. We thus investigated the extent of DV regionalization and its related boundaries by systematically searching for gene expression patterns with specific DV domains. We found that most of the examined patterns could be classified into three groups (Figs 3–9): (i) peripheral genes, with expression restricted to the periphery of the neural plate (future topologically dorsal or alar zone; *Six3/6*, *Lhx2/9b*, *Zic*, *Msx*, *Pax2/5/8*, *Pax3/7*, *Nova*); (ii) internal genes, with expression domains respecting the former peripheral longitudinal zone (*Pou3f*, *Sim*, *FoxD*, *Meis*, *Lef*, *Lhx1/5*, *Hox3*, *Hox6*, *FoxB*); and (iii) pan-DV genes, expressed across both aforementioned domains (*Otx*, *Gbx*, *Fezf*, *Irx*, *Pax4/6*, *Six3/6*, *Nkx2*.*2*, *Meis*, *Rx*, *Hox1*, *Wnt3*, *Wnt7*, *Nova*, *Ebf*). A few markers were ascribed to two of these categories since they have DV expression subdomains that differ depending on the AP partition in which they are expressed (see below and Fig 9). Altogether, these patterns suggest the existence of continuous basal and alar plate zones that extend longitudinally throughout the amphioxus neural tube primordium. As in vertebrates, the right and left moieties meet frontally around the rostral end of the floor plate (Fig 10A), as clearly exemplified by the alar expression of *Lhx2/9b* (Fig 6C′ and 6C′′).

### Conserved *Otx* and *Gbx* expression patterns define a primary AP partition

In all vertebrates, dynamic antagonistic expression of *Otx* (rostral) and *Gbx* (caudal) in the neural plate eventually reaches an equilibrium at the caudal end of the midbrain, defining the MHB (Fig 3A and 3D; [5,83–88]). Clonal labeling studies performed in frogs at the 64 blastomere stage showed that this is the earliest detectable brain transverse boundary [89]. A comparable boundary is also present in amphioxus, aligned between the first and second somites [37], which we further corroborated at the 7-somite neurula stage in *B*. *lanceolatum* (Fig 3B–3F). Accordingly, it was suggested that the first intersomitic limit of amphioxus roughly marks the genetic homolog of the MHB of vertebrates [37,52,53,58,90]. Thus, it can be postulated that these early expression domains in both lineages define the boundary between a rostral *Otx*-positive “archencephalic prototagma” (ARCH; Fig 3J and 3M) and a caudal *Gbx*-positive “deuteroencephalic prototagma” (DEU; Fig 3J and 3M). In addition to *Gbx*, several other amphioxus genes show specific expression within DEU at this stage, abutting rostrally the ARCH–DEU boundary (*Wnt3*, *Wnt7*, *FoxB*, *Pax2/5/8*, and *Msx*; Figs 6G–6H′′ and 7E–7G′′).

### The archencephalon is regionalized anteroposteriorly into the HyPTh and DiMes regions

We previously showed that the ARCH domain can be subdivided anteroposteriorly based on *Fezf* and *Irx* expression [36]. In both vertebrates and amphioxus, *Fezf* genes are expressed in the rostral-most part of the CNS at early neural tube stages, creating an anterior subdomain within the *Otx*-positive territory (Fig 3G–3I) and thus leaving a gap between the caudal end of their expression and the start of that of *Gbx* in DEU (Fig 3K and 3L). On the other hand, *Irx* genes are expressed within this gap, abutting rostrally with *Fezf* and extending posteriorly into the *Gbx*-positive DEU tagma (Fig 3K–3O). Studies in *Xenopus*, zebrafish, and mice, comparing *Fezf* and *Irx* expression patterns with fate mapping data, have shown that the transverse *Fezf*-*Irx* interface marks the prethalamo–thalamic boundary where the ZLI will develop [21,91–94]. Based on the expression patterns observed in the 7-somite amphioxus neurula, we accordingly defined a rostral *Fezf*-positive HyPTh and a caudal *Irx*-positive DiMes intercalated between the *Fezf-*positive and *Gbx-*positive domains (Fig 3K–3O). Remarkably, several genes, including *Pax4/6*, *Six3/6*, *Pou3f*, and *Sim*, are expressed specifically or most strongly within the DiMes (Fig 4, see also Fig 8A–8C′′), supporting the distinct identity of this region. Moreover, other genes in addition to *Fezf* appear restricted to HyPTh (e.g., *Rx* throughout it, Fig 5B and 5B′′, and *FoxD* in its basal plate subdomain, Fig 5C and 5C′′; see also Figs 8D–8E′′ and 9) or have distinct expression subdomains within HyPTh (e.g., *Nova*, S2C and S2′′ Fig). On the other hand, other markers, such as *Ebf*, are expressed caudally to the *Fezf/Irx* limit, similarly to the three *Irx* genes (*IrxA-C*)(Figs 3N, 3O, 4G, 4G′, 6A and 6B′′).

Triple fluorescent in situ hybridization and confocal 3-D reconstruction show that the *Fezf*-positive HyPTh, the *Pax4/6*-positive DiMes and the *Gbx-*positive RhSp domains abut sharply one another. Interestingly, the intermediate domain, DiMes, is very small, consisting only of two rows of cells along the AP dimension (Fig 8A–8B′′). Analogous fluorescent in situ hybridization comparison of *Fezf*, *Six3/6*, and *Gbx* patterns shows that *Six3/6* is also strongly expressed in the DiMes compartment (Fig 8C–8C′′). The *Fezf* and *Gbx* markers are expressed with similar mutual relationships also at the 4/5-somite (early neurula) stage (S3A–S3B′ Fig), leaving an expression gap where weak *Six3/6* signal can already be detected (S3E–S3F′ Fig). Therefore, both the ARCH/DEU limit and the HyPTh and DiMes subdivisions within ARCH are established very early in amphioxus CNS development.

### The HyPTh is divided into three AP domains

We next sought to identify further AP molecular partitions within the HyPTh and DiMes forebrain domains of the 7-somite neurula. Unlike the DiMes, for which we could not identify any molecular subdivision, eight examined markers showed restricted expression domains within HyPTh, sometimes limited to either alar or basal regions. Their combined pattern is consistent with the existence of three AP subdivisions within the HyPTh, which we termed Rostral HyPTh, Intermediate HyPTh and Caudal HyPTh (Rostral-HyPTh, Interm-HyPTh, Caudal-HyPTh; Fig 10A; see Discussion for possible homology relationships with partitions in the vertebrate forebrain). For instance, the expression of six rostral markers (*Nkx2*.*2*, *Nova*, *Meis*, *Pou3f*, *Lef*, *Lhx2/9b*) appears across Rostral-HyPTh and Interm-HyPTh, but seems to respect a transverse double row of cells that lie anterior to the *Irx*-expressing DiMes; topologically, this caudal negative domain of HyPTh (Caudal-HyPTh) would correspond in vertebrates to the primordium of the prethalamic region (Fig 10A). This partition can be visualized as a gap of negative labeling, e.g., by double in situ hybridization for *Meis* and *IrxC* or for *Nkx2*.*2* and *IrxB* (Figs 5G–5G′ and 8F–8F′′, respectively).

While *Nkx2*.*2* and *Nova* signals are present at both alar and basal levels of Rostral-HyPTh and Interm-HyPTh (but not in the corresponding part of the floor plate; Fig 5D–5D′′ and S3C–S3C′′ Fig; see schematic details of expression in Fig 9), *Pou3f*, *Meis*, and *Lef* expression is restricted to the local basal region (also respecting the floor plate; Figs 4F–4F′′, 5F–5F′′ and 5H–5H′′, respectively), and *Lhx2/9b* expression appears selectively in the peripheral alar region (Fig 6C–6C′′). *Six3/6* is the only studied marker restricted to the Rostral-HyPTh, specifically in the alar plate (Figs 2J, 4D–4D′′ and 8C–8C′′; similar expression of *Six3/6* in the anterior-most part of the neural plate was also reported in *B*. *floridae* [81]). On the other hand, *Zic*, which is a well-known marker of the alar and roof plates in the CNS of vertebrates [95], is expressed throughout the presumptive alar plate of the HyPTh, but its expression is significantly stronger at the Caudal-HyPTh, showing decreasing signal towards the rostral alar parts of the HyPTh complex (S2G–S2G′′ Fig). In contrast to the major HyPTh and DiMes partitions, the three HyPTh molecular subdivisions are not fully established at the 4/5-somite stage (S3 Fig).

### Anteroposterior regionalization of the amphioxus DEU domain

Numerous amphioxus genes have been previously reported to show iterative expression domains within the DEU, suggesting the existence of characteristic subdivisions within this partition ([96–99], and see S1 Table). Consistent with these studies, we identified several molecular AP partitions within the rostral-most subdomain of DEU, referred to here as the RhSp, which roughly ends caudal to the fifth somite at the 7-somite neurula stage (Figs 9C and 10A). *Gbx*, *Wnt3*, *Wnt7*, and *FoxB* appear selectively expressed throughout the RhSp; all of them abut rostrally the DiMes/RhSp boundary, and their expression domains end at different caudal levels, either coinciding with the end of somite five or extending further caudalwards (Figs 3E, 3F, 6G–6G′′, 6H–6H′′ and 7E–7E′′). *Gbx*, *Wnt3*, and *Wnt7* occupy both alar and basal regions (but not the floor plate), as previously described [37,100,101], while *FoxB* is restricted to basal areas (Fig 7E; see also [102]). *Hox1*, *Hox3*, and *Hox6* genes are also expressed along alar and basal parts of the RhSp, with rostral expression borders that correspond with the intersomitic limits S3/S4, S4/S5, and S5/S6, respectively (Fig 7A–7C′′; see also [53–57]). As mentioned above, these molecular partitions are complemented by patterns of iterated spots with negative intervals, which can be aligned with the center (*Lhx1/5* and *Pou3f*, Figs 6E–6E′′ and 4F–4F′′) or posterior half of the somites (*Nova*, S2C–S2C′′ Fig) or the inter-somitic boundaries (*Pax3/7*, S2E–S2E′′ Fig). In the case of *Pax2/5/8*, patches are less well defined, particularly caudally, where they become nearly continuous (Fig 7F–7F′′). Finally, some genes show isolated spots of expression located at different positions within the RhSp AP subdivisions: *Msx* (Fig 7G–7G′′), *Meis* (Fig 5F–5F′′), *Zic* (S2G–S2G′′ Fig), *Nkx6* (Fig 7D–7D′′), and *Otp* (Fig 6F–6F′′), sometimes correlating with the prospective position of the future pigmented photoreceptor spot.

### Experimental suppression of the ZLI and IsO organizers in vertebrates alters di-mesencephalic patterning and generates a remnant that resembles the amphioxus DiMes

**Fig 11:**
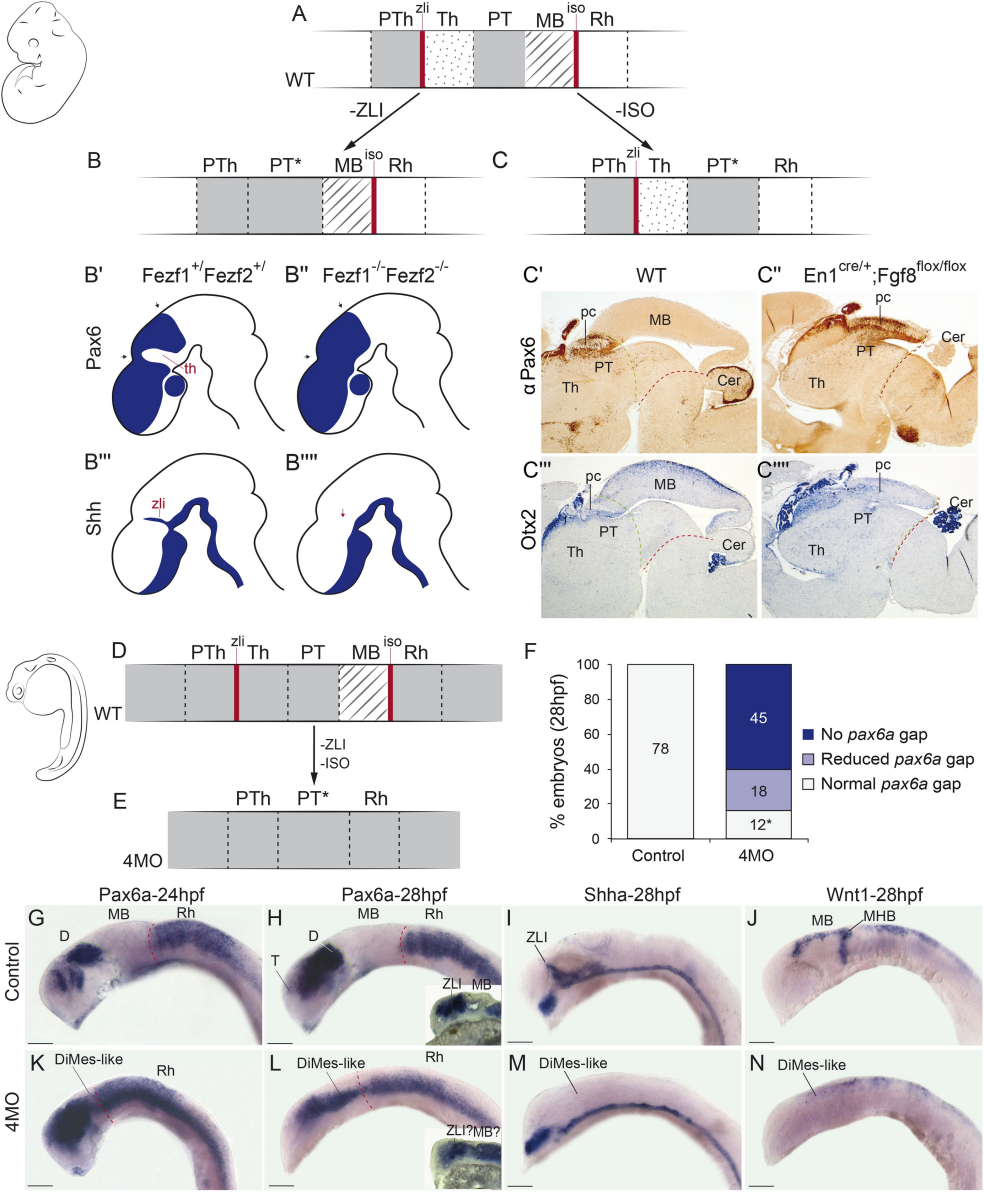
Experimental disruption of secondary organizers in vertebrates results in a Di-Mesencephalic primordium (DiMes)-like remnant. **(A-C)** A schematic representation of mouse *Pax6* neural expression pattern (solid grey) in wild-type condition (WT) **(A)** and abolishing the function of the zona limitans intrathalamica (ZLI) **(B)** or isthmic organizers (IsO) **(C)**. (**B′-B′′′′**) Drawings adapted from the results of Hirata et al. 2006 upon ZLI abrogation during mouse development. **(C′-C′′)** Immunohistochemical detection of αPax6. (**C′′′-C′′′′)** In situ hybridizations for *Otx*. **(C′)** and **(C′′′)** are WT expression domains of *Pax6* and *Otx*, whereas **(C′′)** and **(C′′′′)** are conditional *En2*-*Fgf8* knockout (KO) mice. (**D,E)** Schematic representation of *pax6a* gene expression (solid grey) in zebrafish embryos in WT **(D)** or quadruple morpholino knockdown (4MO) of *otx1a*, *otx2*, *eng2a*, and *eng2b* **(E)** conditions. (**F)** Quantification of the phenotypes observed upon 4MO treatment. All treated embryos showed a reduction in the size of the gap, even those scored as normal (indicated by an asterisk). Embryos with “reduced *pax6a* gap” showed only a very small expression gap, often with weak *pax6a* expression in it. Embryos with “no *pax6a* gap” had a continuous expression of the gene. (**G-N)** Expression patterns of key genes in WT **(G-J)** or 4MO **(K-N)** embryos. Insets in H and L showed sagittal sections of a different representative embryo. Sections of the indicated embryos. Abbreviations: Cer, cerebellum; D, diencephalon; Rh, rhombencephalon; pc, posterior commissure; pTh, prethalamus; Th, thalamus; PT, pretectum; MB, midbrain; MHB, midbrain–hindbrain boundary; T, telencephalon. Anterior is to the left. Scale bar = 100 μm.

A major implication of our comparison of the overall CNS genoarchitecture between amphioxus and vertebrates is that the small amphioxus *Pax4/6*-positive DiMes corresponds topologically to the large vertebrate region comprising the thalamus, pretectum, and midbrain (Fig 10A–10B′′, see Discussion). Patterning of this territory in vertebrates occurs under the dual control of the secondary brain organizers (ZLI and IsO, see Introduction), which induce the molecular subdivision and differential growth of an initially *Pax6*-positive primordium. Among other effects, these organizers inhibit the expression of *Pax6* at the two ends of the territory so that *Pax6* signal becomes restricted to the caudal pretectum (p1) and the epithalamus (dorsal-most part of p2)(Fig 11C′). Accordingly, we hypothesized that the small size and the lack of internal regionalization of the DiMes, particularly with respect to *Pax4/6* expression, may be, at least in part, related to the absence of ZLI-like and IsO-like effects in amphioxus.

To gather support for this hypothesis, we turned first to loss-of-function transgenic mouse lines in which either the ZLI or the IsO are absent. Double *Fezf1*^-/-^*Fezf2*^-/-^ mutants [103] lack the ZLI organizer and largely lose the molecular identity of the alar thalamic field, displaying expanded expression of the pretectal markers *Pax6*^+^ and *Ebf1*^+^ (Fig 11B and 11B′–11B′′′′); the midbrain was not altered in these mice. We also studied *En1*^*cre/+*^*; Fgf8*^*flox/flox*^ mice (see Materials and Methods) in which the IsO is deleted across the MHB [104]. The resulting phenotype showed a reduction of the AP dimension of the pretecto-mesencephalic region down to one-third of its normal size and an abnormal caudal expansion of PAX6 immunoreaction (as well as of the posterior commissure), suggesting a lack of differential specification of the midbrain versus the pretectum (Fig 11C and 11C′–11C′′′′); in this case, the thalamus seemed normal.

Next, we tried to eliminate both the ZLI and the IsO together in zebrafish, using quadruple morpholino (4MO) treatment against *otx1a*, *otx2*, *eng2a*, and *eng2b*. Although effects in neural progenitors of other areas cannot be ruled out, double morpholinos against *otx1a* and *otx2* were successfully used previously to specifically abolish the ZLI [105], while double morpholino treatment against *eng2a* and *eng2b* caused the loss of the IsO [106], and expression of *pax6a* throughout the midbrain remnant [107]. Strikingly, the normal *pax6a*-negative gaps corresponding to the alar plate of the midbrain and diencephalic thalamus were abolished or severely reduced in nearly all (84%) 4MO specimens tested (Fig 11F; *n* = 75, *p* = 3.84 × 10^−31^, one-sided Fisher Exact test), often resulting in a continuous expression of *pax6a* between the rostral conserved part of the forebrain and the hindbrain (Fig 11G, 11H, 11K and 11L and sagittal sections in insets in Fig 11H and 11L). Supporting the effective suppression of the two organizers in this experiment, we observed disappearance of the dorsal ZLI spike expression of *shha* and of the MHB-related transverse band of *wnt1* expression (Fig 11D, 11E, 11H, 11I, 11L and 11M). In addition, all 4MO embryos showed a significant reduction of the zebrafish DiMes-like remnant at 28 h post fertilization (hpf) compared to the controls (Fig 11G′ and 11K′).

## Discussion

Previous studies using gene markers have shown that the developing amphioxus CNS displays marked spatial molecular heterogeneity at different developmental stages ([61,97,102,108–112] and see S1 Table). However, most of these studies focused on individual genes across diverse developmental time points, making it difficult to precisely compare the relative positions of their expression patterns and to elaborate a unified map. In this study, we built a comprehensive genoarchitectonic model of the amphioxus developing CNS by mapping many gene markers at a single developmental stage, allowing homochronic comparisons of gene expression patterns. We used 48 gene markers whose orthologs have known expression patterns in the developing vertebrate CNS and a well-established morphological interpretation within an explicit Bauplan (the updated prosomeric model [2,4]). We focused primarily on the 7-somite neurula stage, in which the majority (43/48, 89.6%) of the examined gene markers were expressed in the incipient neural tube. By the combination of these gene markers, we propose a genoarchitectonic model that, although simpler than that of vertebrates, reveals an unexpected complexity of molecularly defined regions in the developing amphioxus CNS, comprising at least nine AP and three distinct DV partitions (Fig 10). This model provides a base for future exploration of the development of the amphioxus CNS at earlier and later developmental stages and should help in elucidating the ontogenetic origins of larval and adult brain structures. Furthermore, it allows direct topological comparisons with equivalent genoarchitectonic models in vertebrates, since both lineages develop their CNS through homologous neural plates, providing more solid evidence for homology assignments between topologically equivalent regions than mere similarities of relative gene expression patterns.

### Integrative genoarchitectonic model of the amphioxus incipient neural tube

Consistent with previous results [37,59,64], our data show that the incipient amphioxus neural tube is molecularly divided anteroposteriorly into a rostral archencephalic (ARCH) and a caudal deuterencephalic (DEU) portions from very early stages, similarly to vertebrates (Fig 10A and 10B). Traditionally, three main AP divisions are defined in the vertebrate ARCH (Fig 1): the secondary prosencephalon (encompassing hypothalamus plus telencephalon), the diencephalon, and the midbrain. On the contrary, the ARCH of amphioxus shows only two main divisions, which we termed DiMes and HyPTh (Fig 10B). DiMes is a small caudal region consisting of two rows of cells that occupies the topological position corresponding in vertebrates to the midbrain and the two diencephalic segments that lie caudal to the ZLI organizer; no internal subdivisions were detected within DiMes. In contrast, the HyPTh encompasses three molecularly distinct segments: a relatively large, bipartite, putative hypothalamus-homolog region (where neither telencephalic nor optic vesicles are present [113]) plus a caudal region that occupies the topological position corresponding to the vertebrate prethalamus. In the case of the DEU, its rostral portion, referred to here as RhSp primordium, may represent a field-homolog of the vertebrate hindbrain, and shows a number of gene expression patterns that configure periodic segment-like territories (Fig 9C).

Notably, these major AP partitions of the developing CNS are mirrored by molecularly defined subdivisions in the underlying axial mesoderm. Indeed, we provide evidence that the distinct molecular entity at the rostral tip of the amphioxus notochord may be homologous to the vertebrate prechordal plate, being thus essentially different from the notochord proper that underlies the brain floor plate (Fig 2). This potentially prechordal region lies topologically rostral to the HyPTh (not **under** it), as occurs with the prechordal plate in vertebrates [2,4], and is characterized by the absence of *Hh* and *Nkx6* and the specific expression of *Six3/6*, which is also characteristic of the vertebrate prechordal plate [24]. Therefore, it is possible that this special notochord-looking region—which also shows unusual proliferation and rostralward growth [70,114]—may correspond to a variant prechordal plate homolog and/or plays partly equivalent signaling functions to this structure in amphioxus, despite the absence of some key vertebrate prechordal markers (*Gsc*, noggin, and chordin [79,115]).

Finally, regarding DV patterning, multiple markers provide extensive evidence for continuous molecularly distinct floor, basal, and alar zones throughout the length of the incipient neural tube (Figs 9 and 10). Although we did not find selective markers for the roof plate, it is possible that these may exist at later stages, upon neural tube closure. Consistent with the idea that the alar plate and roof plate are not differentially specified in amphioxus at these stages, orthologs of several vertebrate neural plate border makers (e.g., *Pax3/7*, *Msx*, and *Zic*) were found to be expressed broadly in the alar plate (Fig 9; observed also in *B*. *floridae* [116]).

### Possible homology relationships between HyPTh partitions and vertebrate forebrain neuromeres

According to the updated prosomeric model [4], the nontelencephalic part of the vertebrate secondary prosencephalon can be subdivided into two main neuromeres: terminal (THy, hp2) and peduncular (PHy, hp1) hypothalamic prosomeres. In addition, THy includes a specialized rostral-most median part extending dorsoventrally, the acroterminal area [2,4,117](Fig 10C′′). This molecularly distinct domain produces a number of specialized formations along the DV axis, including the alar preoptic lamina terminalis, the optic chiasma, the eye vesicles, the basal median eminence, and the neurohypophysis.

In amphioxus, HyPTh represents a relatively large, molecularly distinct forebrain region lying rostral to the DiMes. This domain has specific expression of *Fezf* throughout (Fig 9), which is also absent caudal to the ZLI limit in vertebrates [93,103,118]. Our analysis suggests that there are three molecularly distinct AP subdivisions within the amphioxus HyPTh, which we termed Rostral-HyPTh, Interm-HyPTh, and Caudal-HyPTh. By direct topological ascription, these might correspond, respectively, to the transverse THy (including a rostromedian acroterminal region) and PHy hypothalamic segments and a prethalamus-like segment next to the DiMes.

*Six3/6* was the only studied marker that selectively labeled Rostral-HyPTh. Remarkably, in mice, *Six3* is expressed extensively dorsoventrally across the alar and basal zones of THy (including the acroterminal area), whereas *Six6* signal is restricted to a ventral suprachiasmatic part of the THy acroterminal alar plate, but none of them are expressed at PHy [2,117]. These data support a genetic equivalence between the Rostral-HyPTh and THy, in addition to their topological correspondence. Moreover, amphioxus develops in its acroterminal region (orange domain in Fig 10C and 10C′) a median primordial eye patch and, ventral to it, a median group of “infundibular cells” [90], which are located above the most anterior floor plate cells (gray cells in Fig 10C) and might represent a homologue of the vertebrate neurohypophysis. As mentioned above, in vertebrates, both the eyes and the neurohypophysis develop from the acroterminal area [2], further supporting the homology of vertebrate and amphioxus acroterminal domains and thus of Rostral-HyPTh and THy (Fig 10C–10C′′).

In the case of the Caudal-HyPTh primordium, its topological position, lying directly rostral to the *Fezf*-*Irx* boundary, provides grounds to suggest field homology with the vertebrate prethalamus. Importantly, previous studies indicate that *Fezf* genes are essential to specify the prethalamic domain in vertebrates; however, unlike regions within the vertebrate DiMes counterpart (see below), this specification is independent of the ZLI organizing activity and occurs prior to its formation [93,103] and is thus compatible with the amphioxus scenario at the examined stage. Nonetheless, it should be noted that, although more weakly expressed, the presence of *Rx* expression in Caudal-HyPTh (absent in the prethalamus of vertebrates [118]), suggests the alternative possibility that this partition may represent a primordium homolog to both the peduncular hypothalamus and prethalamic region.

### Close developmental and evolutionary relationship of thalamus, pretectum, and midbrain

One of the most striking implications of our results is that the small, *Pax4/6*-positive DiMes of amphioxus corresponds topologically to the region comprising the vertebrate thalamus, pretectum, and midbrain (Fig 10). While this area is not subdivided in amphioxus and consists only of two cell rows at the neurula stage, the equivalent vertebrate region shows three major partitions and extensive cell proliferation. These partitions in vertebrates originate during development as a consequence of the action of the secondary brain organizers on a *Pax6*-positive primordium. In particular, *Shh* signaling from the ZLI is crucial for the specification of the thalamus [6–8,119], and *Fgf8* and *Wnt1* expression from the IsO are necessary for proper midbrain specification and differential caudal growth [5,10,12,13,120–123]. Moreover, due to the action of these organizers, the expression of *Pax6* in this primordium is mainly restricted to the pretectum and the epithalamus and becomes absent in the ventricular zone of the thalamus and midbrain (Fig 11) [124,125].

Therefore, altogether, these data suggest that the vertebrate thalamus, pretectum, and midbrain share a common origin, both ontogenetically (from an early and transient *Pax6*-positive area found between the prospective ZLI and IsO levels) and phylogenetically (homologous to the amphioxus DiMes region). This hypothesis has two major implications for our understanding of the vertebrate brain Bauplan and its evolutionary origins. First, it implies that two of the diencephalic prosomeres—pretectum (p1) and thalamus (p2)—are more evolutionarily related to the midbrain than they are to the third diencephalic prosomere—the prethalamus (p3)—which would, in turn, be more related with the secondary prosencephalon (see previous section). That is, the diencephalon proper would be neither an evolutionarily nor an ontogenetically primordial subdivision of the vertebrate brain. This striking implication is further supported by the differential responses of these regions to experimental manipulation of the organizers and their associated signaling molecules. Chicken-quail heterotopic grafts of the ZLI, as well as focalized ectopic expression of *SHH* using beads in chicken embryos, show that only pretectum and midbrain, but not the prethalamus, are competent to be re-patterned to a thalamic fate [6,7,119]. Similarly, quail-chick, rat-chick, or mouse-chick heterotopic grafts of the IsO generate an ectopic midbrain in pretectal and thalamic regions, but never in the prethalamus and secondary prosencephalon [126–129]. That is, thalamus, pretectum, and midbrain have similar developmental potentials that are not shared by the prethalamus. In fact, our hypothesis provides a plausible ontogenetic explanation that has long been missing for these intriguing observations, underscoring its explanatory power.

A second major related implication of our hypothesis is that the vertebrate thalamus, pretectum, and midbrain jointly share altogether a common ancestor with the amphioxus DiMes. Since neither *Hh* nor *Fgf8* and *Wnt1*, the key morphogens involved in ZLI and IsO activity, respectively, are expressed at the corresponding topological positions in amphioxus [40,69,130–132], it is plausible to speculate that vertebrate thalamus, pretectum, and midbrain partitions may have emerged evolutionarily from an ancestral *Pax4/6*-positive DiMes-like region concomitantly to the evolution of the ZLI and IsO brain organizers as orthogonal signaling centers. Alternatively, the undivided, small amphioxus DiMes may represent an evolutionary simplification upon the loss of the organizers [47,48], if they were already patterning the neural plate-derived CNS of the last common ancestor of chordates. Irrespectively, a major prediction of both evolutionary hypotheses is that suppression of the organizers during vertebrate development should result in a (relatively) homogeneous, smaller, undivided, and fully *Pax6*-positive region lying between recognizable prethalamus and hindbrain, as we observed in mouse and zebrafish embryos with suppressed ZLI and/or IsO (Fig 11, and see also [103,107,133–136]). Although the converse experiment—the induction of ectopic organizers in amphioxus—is still not technically possible, future methodological developments could allow assessing if and how the DiMes may respond to these morphogens.

Finally, an independent line of evidence supporting the functional homology between the amphioxus DiMes and the corresponding vertebrate regions comes from the retinal projections in the two lineages. In vertebrates, primary eye projections target mainly the midbrain (optic tectum/superior colliculus), while secondary eye projections target mainly the pretectum and thalamus and, to a lesser extent, prethalamus and hypothalamus [137]. In amphioxus, projections from the single frontal eye have recently been mapped to a *Pax4/6*-positive region in the four gill slit larval stage [138], which likely corresponds to a DiMes derivative based on its topological position and *Pax4/6* expression.

## Concluding remarks

Our comprehensive genoarchitectonic model of the developing amphioxus CNS at mid-neurula stage sheds new light onto the origins of the vertebrate brain. First, it shows that the basic blueprint of the vertebrate brain Bauplan was already present in the last common ancestor of chordates. The major AP and DV partitions identified in amphioxus have direct topological correspondence with vertebrate counterparts, even though these may be further elaborated in vertebrates. Such is the case of the eye vesicles and the telencephalon developing as alar expansions of a HyPTh-like region or the growth and regionalization of a DiMes-like region into thalamus, pretectum, and mesencephalon. Secondly, it highlights the importance of the evolution of secondary organizers in the gain or loss of brain partitions. Thirdly, it allowed us to propose novel homologies between amphioxus and vertebrate structures, such as the acroterminal hypothalamic area and the prechordal plate. Finally, it casts doubts on the relevance of the classic separation between forebrain and midbrain in vertebrates from an evolutionary and developmental perspective, suggesting that a redefinition of the main AP regions into which the vertebrate brain is classically divided (forebrain, midbrain, and hindbrain) could provide a better conceptual framework to understand the origins of the vertebrate brain.

## Materials and methods

### Ethics statement

All animal work in this study has been conducted following the Spanish and European legislation. Adult fish were only used to obtain eggs through natural mating (ethical committee approval number: 635/2014). All mouse experiments were performed according to protocols approved by the Universidad Miguel Hernandez OEP committee (UMH.IN.EP.01.13) and Conselleria Generalitat Valenciana (2014/VSC/PEA/00055). Chicken experiments were performed according to protocols approved by the ethical committee from the University of Murcia (137/2015).

### Gene annotation and cloning

For all the previously annotated genes in the *B*. *floridae* genome, primer pairs were designed to span the full-length coding sequence when possible. A liquid cDNA library from different developmental stages of the European amphioxus (*B*. *lanceolatum*) was screened by PCR using *B*. *floridae* specific primers. For previously unannotated genes, we performed tBLASTN searches in the *B*. *floridae* JGI v1.0 genome, using the aminoacidic sequences of the vertebrate orthologs. The corresponding genomic sequences were retrieved and a gene model was predicted by GeneWise2 and GeneScan, as previously described [139]. Cloned *B*. *lanceolatum* mRNAs used for in situ hybridization are available in S2 Table.

### Amphioxus embryo collection, whole-mount in situ hybridization, and histology

Ripe adult amphioxus specimens were collected in Argelès-sur-mer, France. Spawning was induced as previously described [140] in a dry laboratory in Barcelona, Spain. After in vitro fertilization, embryos were cultured at 18 ºC for 15 h or 21 h (4/5 somite and 7 somite stages, respectively) and fixed with 4% PFA in MOPS buffer overnight at 4°C.

Chromogenic whole-mount in situ hybridization was performed as previously described [36] using Nitrobluetetrazolium/bromochloroindolyl phosphate (NBT/BCIP) or BMP purple (Roche) as chromogenic substrate for the final alkaline phosphatase. Following whole-mount in situ hybridization, selected embryos were embedded in a 0.1 M PBS solution with 15% gelatine and 20% sucrose, frozen in isopentane, and sectioned with a cryostat at 12–14 μm-thick. Double-fluorescent in situ hybridizations were performed essentially as nonfluorescent in situ hybridizations, as described in [141] with two extra steps of incubation in 5% NAC and (50 mM DTT, 1% NP40, 0.5% SDS) in PBS1X before the hybridization step.

Dinitrophenol (DNP)-labeled antisense riboprobes were synthesized using DNP-11-UTP labeling reagent (PerkinElmer), and DIG-labeled antisense riboprobes were synthesized using DIG RNA labeling mix (Roche). Labeled riboprobes were detected using anti-DNP-POD (Perkin Elmer) and anti-DIG-POD (Roche) antibodies, and green and red fluorescent signals amplified with TSA -Plus -Fluorescein and Tetrarhodamine systems (Perkin Elmer), respectively.

Images were acquired using a Leica TCS-SPII confocal microscope or a Zeiss Axiophot. Confocal datasets were deconvolved with Huygens Professional version 16.05 (Scientific Volume Imaging, The Netherlands, http://svi.nl), analyzed, and assembled with ImageJ; for panels B and B′ in Fig 8, images were further processed with Imaris (7.2.3, Bitplane AG, software available at http://bitplane.com).

### Fish husbandry, morpholino treatments, and in situ hybridization in zebrafish embryos

Breeding zebrafish (*Danio rerio*) were maintained at 28°C on a 14 h light/10 h dark cycle as described in [142]. To disrupt the ZLI and IsO secondary organizers together, we performed a quadruple transient knockdown using four morpholino-antisense oligomers (MOs) that had been previously described to abolish each of the organizers individually: *otx1a* and *otx2* MO’s for the ZLI [105], and *eng2a* and *eng2b* for the IsO [106]. As injection controls, we used a combination of the two nontargeting MOs that were used in the original articles (a morpholino-sense oligomer against *twhh* (Cont1) [105] and a standard control MO (Cont2) [106]). The combination of experiment or control MOs was injected at the one-cell stage into the yolk at the following concentrations (based on the original sources): *otx1a* (0.25 mM), *otx2* (0.25 mM), *eng2a* (0.5 mM), *eng2b* (0.5 mM), Cont1 (0.5 mM), Cont2 (1 mM). Each embryo was injected with 1.5 nl of the MO mix (injection of 1.0 nl produced similar, yet milder, phenotypes, whereas injection of 2.0 nl resulted in full mortality). Four independent experiments were performed (in different days), injecting approximately 100 eggs per condition and experiment.

Injected embryos were fixed in 4% PFA overnight at 4 ºC and used for whole-mount in situ hybridization as previously described [143]. A subset of stained embryos was cryosectioned, and both sections and whole embryos were mounted in 80% glycerol-PBS and photographed in a Zeiss Axiophot microscope. The full list of probe sequences is available in S3 Table.

### Analysis of gene knockouts in mice

The *Fgf8* conditional mutant was generated by the Gail R. Martin laboratory [120], and the transgenic mouse line expressing *cre* under the *En1* promoter was generated in the Dr. Wolfgang Wurst laboratory [144]. Mutant embryos were generated by crossing double heterozygous males (*En1*^*cre/+*^*; Fgf8*^*flox/+*^) with homozygous *Fgf8*^*flox/flox*^ conditional females. Immunohistochemistry (PAX-6) and in situ hybridization (*Otx2*) in paraffin sections were performed as previously described [145]. The primary PAX-6 rabbit polyclonal IgG antibody was diluted in PBTG (1:500; PRB-278P/Covance). The *Otx2* probe was synthesized as in [86].

### In situ hybridization in chicken embryos

All the procedures involving extraction of brain samples and further tissue processing were done as previously described [146]. Fertilized chicken (*Gallus gallus domesticus*) eggs were bought from a national farm (Granja Santa Isabel; Córdoba, Spain) and incubated at 38 ºC and 65% controlled humidity in a forced draft incubator until the Hamburger–Hamilton stage five (HH5) [147]. Embryos were fixed by immersion in 4% paraformaldehyde in 0.1M phosphate buffered saline (PBS, pH 7.4) during 16 h at 4 ºC. Whole-mount in situ hybridization was done as previously described [146] using probes for *Otx2* and *Gbx2* reported in [24]. *Fezf2* probe was cloned using the following primers: F, GCTACAAACCCTTCGTCTGC and R, GCTCAGGGTCACTTGCTACC.

## Supporting information

S1 Fig Temporal expression of Nk2.1 during amphioxus development. Lateral views (A-F, F’), dorsal views (A’-E’), and schematic drawings (A”-F”) of the neural component of Nk2.1 gene expression pattern from 15 to 36 hours post-fertilization. Anterior is to the left except in F. Somites are indicated using red dotted lines. Scale bar = 50μm.
(TIF)

S2 Fig Additional gene markers used in this study. Other markers with neural expression used in this study in lateral (A-G) or dorsal views (A’-G’), and drawings of the neural component of each gene expression pattern with the relative position of somites (A”-G’). Markers with no expression in the amphioxus developing CNS at this stage are showed in lateral (H-N) and dorsal views (H’-N’). Anterior is to the left. Scale bar = 50μm.
(TIF)

S3 Fig Expression of key gene markers at early neural stage. (TIF)

S1 Table Information on the neural expression of genes used in this study. (XLSX)

S2 Table Probes of amphioxus used for in situ hybridization. (XLSX)

S3 Table Probes of zebrafish used for in situ hybridization. (XLSX)

## Abbreviations

4MO: quadruple morpholino
AP: anteroposterior
ARCH: archencephalic prototagma
CNS: central nervous system
DEU: deuteroencephalic prototagma
DiMes: Di-Mesencephalic primordium
DV: dorsoventral
Evo-Devo: evolutionary developmental biology
hpf: h post fertilization
HH5: Hamburger–Hamilton 5
HyPTh: hypothalamo-prethalamic primordium
IsO: isthmic organizer
KO: knockout
MHB: Midbrain–Hindbrain Boundary
p1: pretectum diencephalic prosomere
p2: thalamus diencephalic prosomere
p3: prethalamus diencephalic prosomere
PHy: peduncular hypothalamic prosomere
RhSp: Rhombencephalo-Spinal primordium
THy: terminal hypothalamic prosomere
ZLI: zona limitans intrathalamica
