# Supplementary figures and images for: Molecular regionalization of the developing amphioxus neural tube challenges major partitions of the vertebrate brain
Source: PLoS Biol. 2017 Apr 19;15(4):e2001573. doi: 10.1371/journal.pbio.2001573 (PMC5396861; doi:10.1371/journal.pbio.2001573)

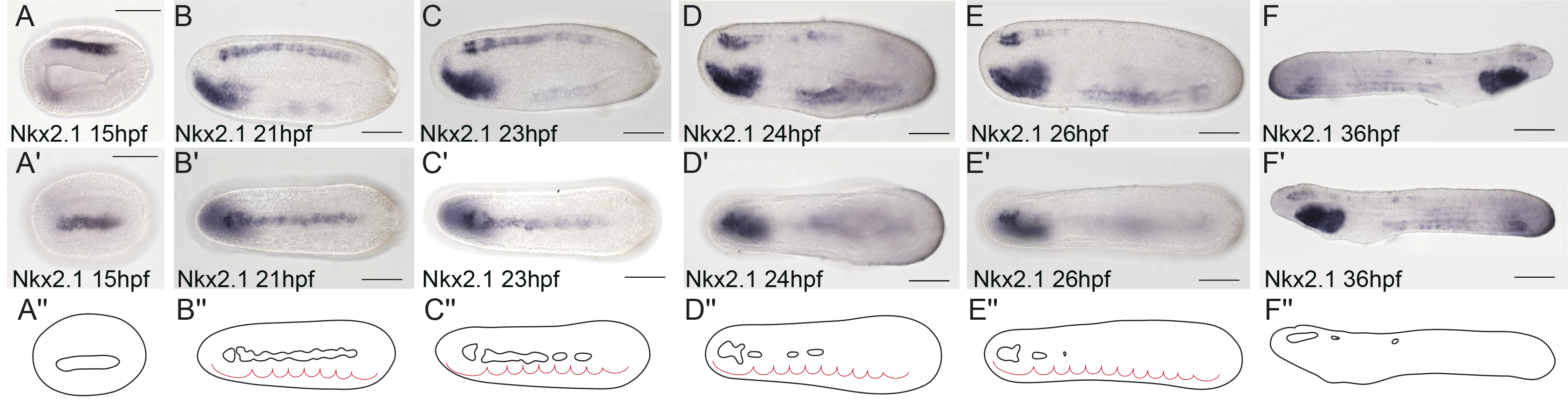

Supplement: S1 Fig — Lateral views (A-F, F’), dorsal views (A’-E’), and schematic drawings (A”-F”) of the neural component of Nk2.1 gene expression pattern from 15 to 36 hours post-fertilization. Anterior is to the left except in F. Somites are indicated using red dotted lines. Scale bar = 50μm. (TIF) [file pbio.2001573.s001.tif]

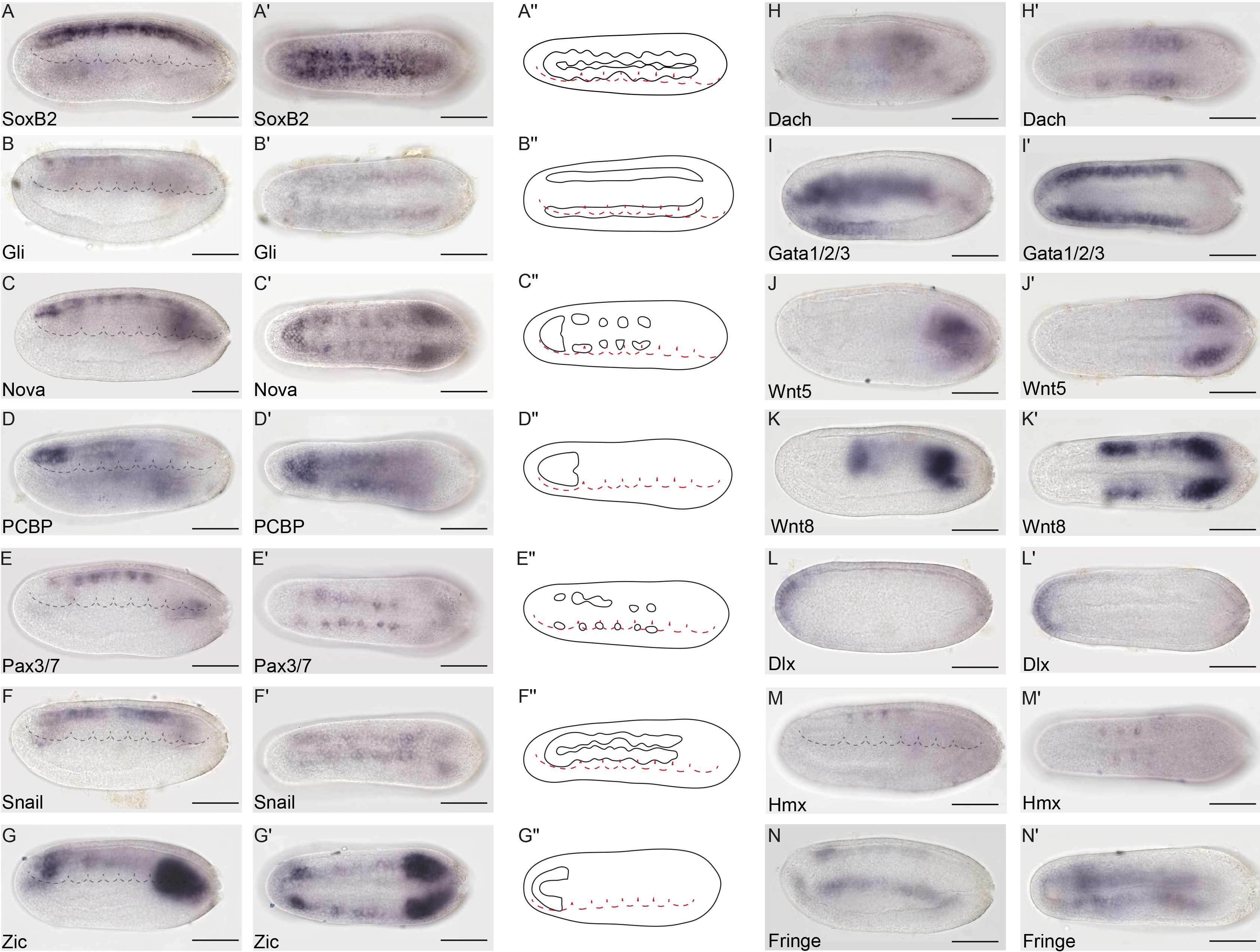

Supplement: S2 Fig — Other markers with neural expression used in this study in lateral (A-G) or dorsal views (A’-G’), and drawings of the neural component of each gene expression pattern with the relative position of somites (A”-G’). Markers with no expression in the amphioxus developing CNS at this stage are showed in lateral (H-N) and dorsal views (H’-N’). Anterior is to the left. Scale bar = 50μm. (TIF) [file pbio.2001573.s002.tif]

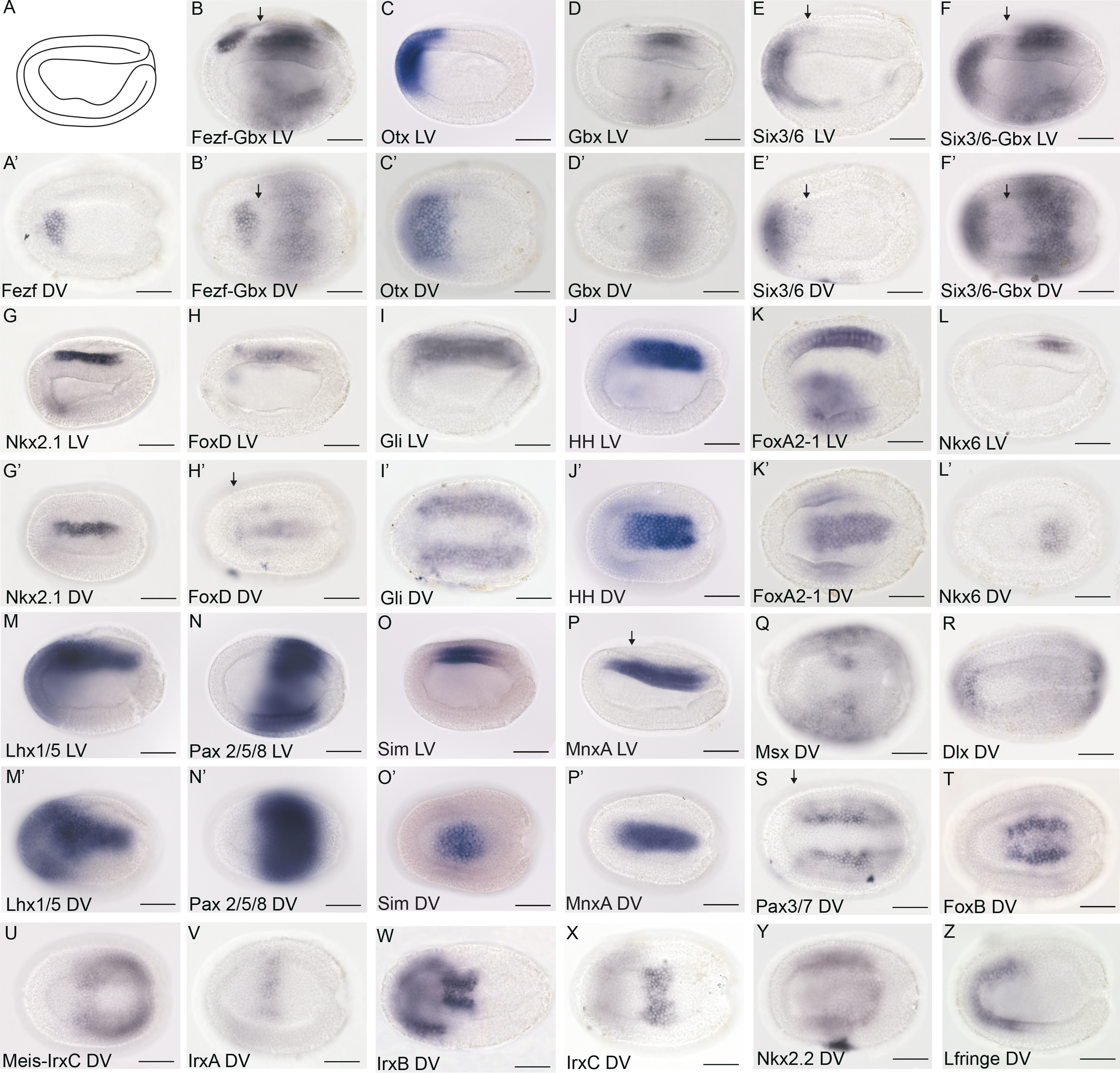

Supplement: S3 Fig — (TIF) [file pbio.2001573.s003.tif]
